# ASICs in PET: what we have and what we need

**DOI:** 10.1186/s40658-025-00717-8

**Published:** 2025-02-13

**Authors:** Vanessa Nadig, Stefan Gundacker, Katrin Herweg, Stephan Naunheim, David Schug, Bjoern Weissler, Volkmar Schulz

**Affiliations:** 1 University Hospital Aaachen, Pauwelsstrasse 30, 52074 Aachen, Germany; 2 Institute of High Energy Physics, Austrian Academy of Sciences, Nikolsdorfer Gasse 18, 1050 Vienna, AT Austria; 3 Hyperion Hybrid Imaging Systems GmbH, Pauwelsstrasse 19, 52074 Aachen, GER Germany; 4 III. Physikalisches Institut B, RWTH Aachen University, Otto-Blumenthal-Straße, 52074 Aachen, GER Germany; 5 Institute for Imaging and Computer Vision, RWTH Aachen University, Kopernikusstraße 16, 52074 Aachen, GER Germany

**Keywords:** Time-of-flight, Application-specific integrated circuits, ASIC, Positron emission tomography, PET, Coincidence resolution time, TOFPET2, PETsys, Weeroc, PETA, NINO, FlexTOT, STiC

## Abstract

**Background:**

Designing positron emission tomography (PET) scanners involves several significant challenges. These include the precise measurement of the time of arrival of signals, accurate integration of the pulse shape, maintaining low power consumption, and supporting the readout of thousands of channels. To address these challenges, researchers and engineers frequently develop application-specific integrated circuits (ASICs), which are custom-designed readout electronics optimized for specific tasks. As a result, a wide range of ASIC solutions has emerged in PET applications. However, there is currently no comprehensive or standardized comparison of these ASIC designs across the field.

**Methods:**

In this paper, we evaluate the requirements posed to readout electronics in the field of PET, give an overview of the most important ASICs available for PET applications and discuss how to characterize their essential features and performance parameters. We thoroughly review the hardware characteristics of the different circuits, such as the number of readout channels provided, their power consumption, input and output design. Furthermore, we summarize their performance as characterized in literature.

**Results:**

While the ASICs described show common trends towards lower power consumption or a higher number of readout channels over the past two decades, their characteristics and also their performance assessment by the developers, producers and vendors differ in many aspects. To cope with the challenge of selecting a suitable ASIC for a given purpose and PET application from the varying information available, this article suggests a protocol to assess an ASIC’s performance parameters and characteristics.

**Conclusion:**

ASICs developed for PET applications are versatile. With novel benchmarks set for the impact of scintillator and photosensor on the time-of-flight performance, the pressure on ASICs to deliver higher timing resolution and cope with an even higher data rate is enormous. Latest developments promise new circuits and improvements in time-of-flight performance. This article provides an overview on existing and emerging readout solutions in PET over the past 20 years, which is currently lacking in literature.

## Introduction

Positron emission tomography (PET) is a functional imaging technique, meaning it can measure the metabolic activity of cells with an unprecedented sensitivity. Established applications are the diagnosis and staging of cancer, neurological diseases, such as Alzheimer’s, and cardiac diseases [1]. Due to recent developments targeting larger axial field of view (aFOV) [2–6] and high time-of-flight (TOF) resolution [7–10], PET extended its classical field of applications and now opens the door to understand organ cross-talk such as gut-heart and gut-brain axis [11], to monitor T cell response, viral load and inflammation in patients [12, 13] and possibly to pave the way to ultra low-dose, screening applications.

In a PET scan, the injected radioactive tracer decays producing a positron. The positron annihilates with an electron to produce two coincident $\gamma$-photons with an energy of 511 keV, which are detected by a gantry of detectors surrounding the patient. The precision with which the system can resolve the difference in arrival times of these two $\gamma$-photons is called TOF resolution or coincidence time resolution (CTR). Each detector element consists of a scintillator, commonly lutetium oxyorthosilicate, ${\hbox {Lu}}_2{\hbox {SiO}}_5$:Ce (LSO) or lutetium yttrium oxyorthosilicate, ${\hbox {(Lu-Y)}}_2{\hbox {SiO}}_5$:Ce (LYSO) [1, 14, 15], a photosensor, nowadays silicon photomultipliers (SiPMs) [16–20], and dedicated readout electronics. Upon detection of a 511 keV $\gamma$-photon, the scintillator produces optical photons detected by the SiPM, which leads to pulse-shaped signals [18, 21]. The digitization of these signals through the readout electronics is required to be highly precise, with the readout electronics adapted to read out the high number of detector channels within the limited gantry space in a PET system [22–26], which becomes even more restricted when combining PET and magnetic resonance imaging (MRI) in a hybrid system [27, 28].

This article aims to provide a thorough overview on the system requirements, the available digitization circuits and a protocol to characterize these, appealing to system engineers and researchers making a selection for highly integrated readout electronics. The article furthermore guides the developers of such circuits with respect to reporting the circuit characteristics and performance to make them appealing for PET applications.

## Requirements for scalable, system-applicable readout electronics

While bench-top setups and some small-scale systems focus on optimizing the TOF performance with custom-designed, ultra-fast, but mostly bulky and power-hungry readout circuits, PET systems rely on scalable and highly integrated electronics to allow the readout of several thousands of sensor channels under rigid space, temperature and power constraints.

### Form factor and packaging

To mitigate challenges imposed by space constraints, readout electronics are required to deliver a high channel density, i.e., a high number of readout channels on an as small as possible package size. The packaging itself might influence how easily it can be integrated into custom-designed electronics. Therefore, if the vendor enables the users to purchase the readout electronics on dedicated printed circuit boards (PCBs) with common connectors, the usage of these becomes independent of its packaging. If the readout electronics can be purchased within a ready-to-use setup package, this may attract a higher number of researchers and allows to use the readout electronics in a plug-and-play manner. The packaging itself can be done by providing a highly integrated circuit on a dedicated PCB chip on board (COB) or by selling individual chips in ball-grid-array (BGA) packaging, which offers a higher flexibility for high-end users envisioning the integration in larger PET systems. Further packaging options are a system in package (SiP) or a flip-chip mount of a single die, called a chip scale package (CSP).

### Power consumption

The power consumption of the readout electronics has to be kept as low as possible to reduce the costs for the power supply infrastructure on the one hand and the respective cooling infrastructure on the other to cope with heat dissipation. This avoids high operation costs for a PET system, which especially is subject to economic efficiency in clinical environments. Additionally, the power consumption aspect becomes key when aiming for compatibility with MRI systems (c.f. Section "MR compatibility"). As standard point-of-load switched-mode power supplies (converting high voltage / low current to the needed low voltage / high current) use ferrite-core inductors, they cannot be used in strong magnetic fields. Thus, normally high currents need to be transported up to the readout electronics, which requires thick cables and wide traces on PCBs. Low current consumption is hence as important as low power consumption. If many different supply voltages are needed, they either need extra wires or local regulators causing additional heat dissipation.

### Sensitivity and data rate requirements

Generally, a high sensitivity, i.e., the ratio between the detected true coincidences and the activity positioned in the field of view (FOV), in the count-rate regime of the application is mandatory for clinical PET. A high sensitivity enables shorter scan times or imaging with a lower radiation dose, while maintaining image quality [11]. A higher sensitivity may be achieved by a larger axial field of view or bringing the PET detectors closer to the organ of interest [2, 29]. The geometry of a PET scanner or setup and the envisioned activity in the FOV pose requirements on the event digitization rate and dead time of the readout electronics. In small-bore scanners, like brain imagers, organ-specific scanners or preclinical scanners, the activity is often in close distance to the PET detectors. With lower distance between activity and PET detector, the $\gamma$-interaction rate increases by the square. The specific implementation of the PET detector in terms of scintillator geometry and its coupling to both the photosensor and the electronic circuits influences the number of channels or the event size, i.e., the number of readout channels fired per event that the readout electronics have to digitize for each $\gamma$-interaction [27]. Furthermore, the readout architecture and trigger scheme plays a crucial role, when evaluating rate capabilities. It is essential to consider whether each sensor channel is connected to a separate input channel of the readout electronics with an individual digitizer [22, 30] or if the output of several sensor channels is multiplexed [31, 32]. In addition, event size and rate may differ if a common trigger is set for a certain amount of sensor channels or if all channels may trigger individually [27]. Preclinical systems, like the SAFIR scanner [33, 34], aim for highest event rates in the order of 1.2 Mcps [34] only limited by the scintillator itself. Clinical scanners on the other hand, e.g., total-body scanners for low-dose applications, generate several orders of magnitude less interactions per scintillator area due to their enlarge FOV. Hence, the event rate capabilities and the behavior when driving the readout electronics towards and above their limits should always be evaluated in the light of the anticipated detector design and system application.

### MR compatibility

Since the 1990 s, PET scanners have been combined with computed tomography (CT) scanners into hybrid imaging systems to register anatomical information and to exploit the possibility of acquiring attenuation maps for the PET image reconstruction [35]. About ten years later, PET detectors were integrated in MRI scanners to enable simultaneous imaging of anatomical information without additional radiation dose and to combine the various image contrasts of MRI with the quantitative molecular imaging of PET [36]. While this reduces the overall dose for the patient, improves workflow in many clinical cases and offers powerful possibilities in research, this combination of modalities is a challenging engineering task that imposes particular requirements on the readout electronics.

**Fig. 1:**
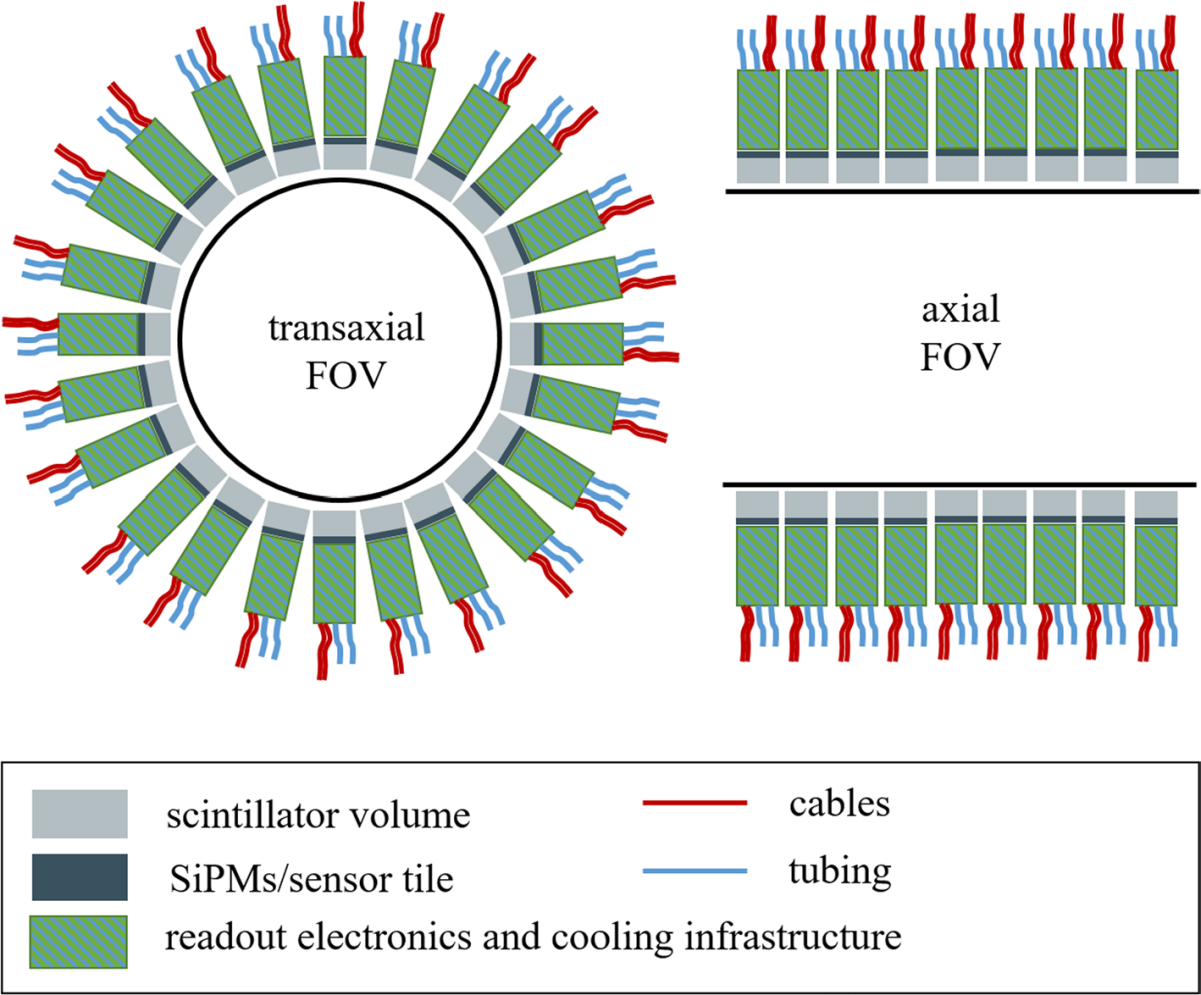
Illustration of the placement of the readout electronics and cooling infrastructure in a standard PET or PET/CT gantry with vast radial space available

**Fig. 2:**
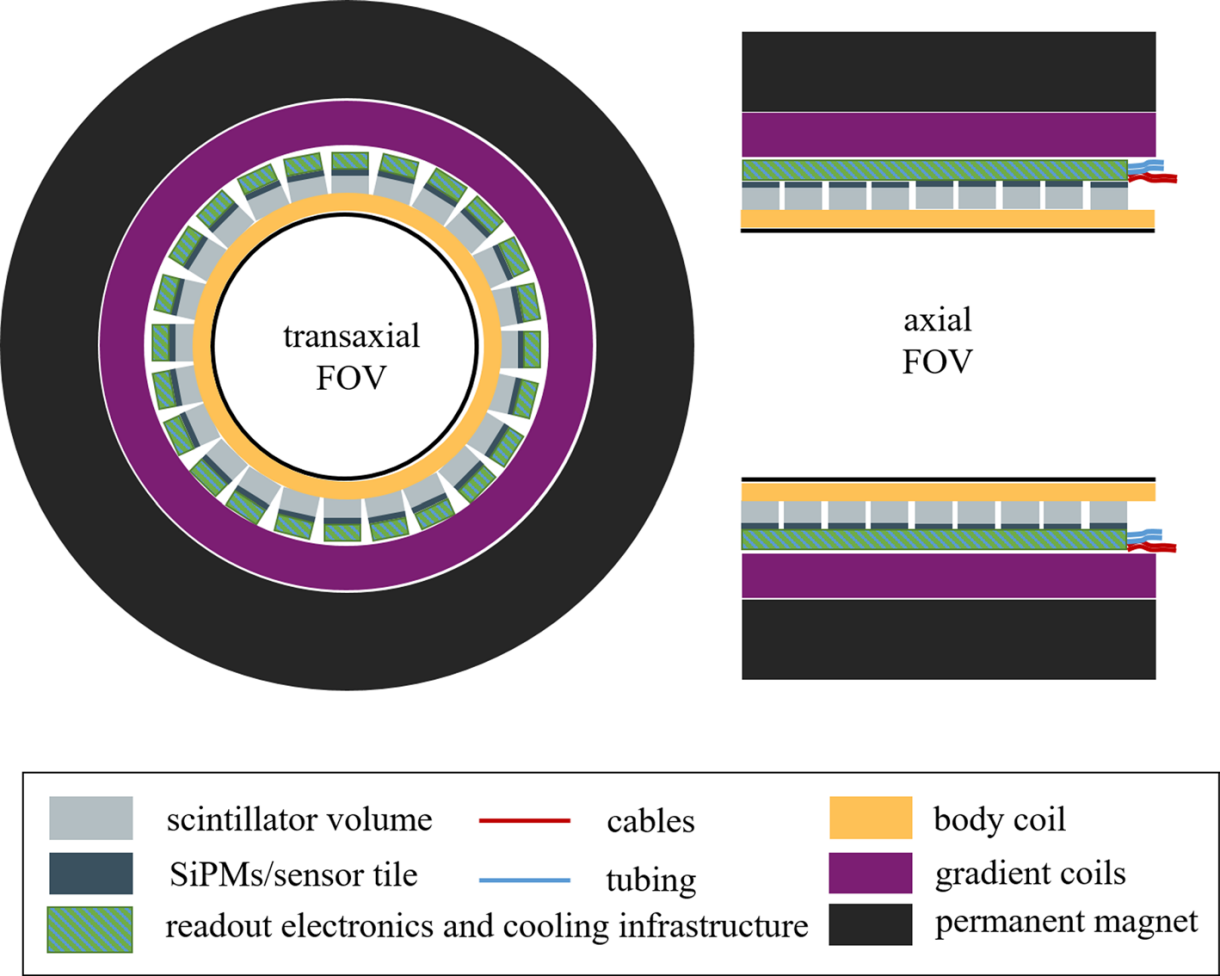
Illustration of the placement of the readout electronics and cooling infrastructure in a PET/MR gantry with restricted radial space available

In PET/CT, the photosensors and readout electronics as well as the cooling infrastructure are placed radially behind the scintillation crystals (c.f. Fig. 1). This enables short-axial FOV units, that can be extended to long aFOV systems by adding multiple copies of such ring-shaped gantries. In PET/MRI, where the PET detectors are normally placed between the gradient coils and the body coil of the MRI scanner (c.f. Fig. 2), the radial space is extremely constrained. Therefore, reducing the infrastructure for electronic circuits as well as cutting back on the amount of communication lines and the space for power supply and cooling is of utmost importance.

The closer the readout and digitization electronics are placed to the FOV of the PET/MRI, the less magnetic material is allowed in their components and supporting circuits. Whereas standard plastic housings are generally not a problem in human whole-body PET/MRI, this might be different in preclinical applications, where everything is packed a lot denser and closer to the MRI FOV. Apart from the restricted available space, the switching gradients of the MRI call for small readout electronics as well. While the powerful radio-frequency (RF) excitation field is usually shielded from the electronics, the gradient switching cannot be shielded and induces voltages in conductive loops and areas, which increase with the loop size and area. Hence, the electronic circuits need to be particularly small. A high number of channels per area, i.e., a smaller form factor, could generally be considered beneficial to support a large detector area. In contrast, a lower amount of readout channels in an even smaller package could be favorable to prevent induction - if the needed infrastructure (supporting circuits, power and communication lines) per package does not obliterate that advantage.

MRI scanners can measure very low signal intensities. In the optimal case, only the thermal noise of the patient should limit the received signal-to-noise ratio (SNR). Spurious signals received from PET detector electronics will directly deteriorate the SNR or show up as artifacts in the MR images [37]. Low emission around the MRI frequency (computed via the gyromagnetic ratio [38] and typically between 64 MHz for 1.5 T and 300 MHz for 7 T), from the readout electronics - and especially from the communication lines as they expand over longer distances - reduce the interference.

In summary, overall power consumption is not the only important factor for PET/MRI applications. Additional parameters include the amount of needed different supply voltages and the current uptake of each voltage (c.f. Section "Power consumption"). Small packages with low amount of ferromagnetic material are advantageous. The number of needed supporting circuits and communication wires should be low. Electromagnetic interference between the MRI scanner and the PET electronics have to be minimal not to deteriorate image quality in both imaging modalities.

### Detector-specific requirements

Considering the digitization of photosensor signals and specifically SiPM signals, the readout electronics need to deal with specific signal shapes and amplitudes. An analog SiPM is an array of single-photon avalanche diodes (SPADs), which are connected in parallel [17, 39]. Each of these SPADs has a basic signal shape of two exponential functions with different rise time ($\tau _r$) and decay time ($\tau _d$) and a defined amplitude A (see Eq. 1, where $t_x$ is the arrival time of an optical photon at the SPAD)[21].

1 $$\begin{aligned} f(t;t_x) = A \cdot \left( e^{-\frac{(t-t_x)}{\tau _d}}- e^{-\frac{(t-t_x)}{\tau _r}}\right) \theta (t-t_x) \end{aligned}$$

In SiPMs, these SPAD signals are summed up to an overall pulse-shaped signal. Thus, the readout electronics in PET applications face signals that depend on the number of SPADs, which break through for an incident $\gamma$-photon, and the time profile of when they break through. This time profile differs drastically depending on the scintillator material. The electronic circuit needs to adapt to the SiPM capacitance, causing a variation in signal shape depending on the number of SPAD and changes in signal bandwidth [21]. Additionally, signals stemming from SiPMs might be deteriorated by various sources of noise, including dark counts, internal and external crosstalk as well as afterpulsing [21]. These signals in the order of one or few SPAD amplitudes can be rejected by specific validation schemes implemented in the readout electronics [22, 40], which determine whether a signal exceeds the amplitude of a single or few single SPAD signals. This can prevent the readout electronics from triggering the complete digitization process, which would impose dead time on the PET system.

#### Scintillation emission/multi-photon detection

The scintillator determines the previously mentioned number of SPAD breakdowns and their time profile by its intrinsic light yield (ILY) and decay time [41]. In multi-photon regimes, a steep signal pulse in the order of several 100 mV is expected [21] in contrast to single-photon regimes where single-SPAD triggers only produce pulses with an amplitude of few dozens of mV [21]. To achieve compatibility with the produced signal, the readout electronics require absolute maximum ratings that match the expected signal amplitudes. The operation point of employed discriminators needs to be calibrated to enable triggering on the rising edge of the signal and generate a timestamp. Additionally, the method to assess the energy of the signal should be adaptable to different signal lengths and shapes (c.f. Section "Signal digitization by electronic circuits"). The most important PET performance parameters in this regime are the CTR and energy resolution, against which the combination of detector and electronics are evaluated. The single-photon time resolution (SPTR), i.e., the ability of the photosensor and readout electronics to resolve the timestamp of single-photon triggers, plays a minor part, but is a feature that might be interesting for some research applications (c.f. Section "Prompt photon emission/Single-photon detection").

#### Prompt photon emission/single-photon detection

In regimes with a lower number of photons emitted, sometimes only single photons, the detector and readout design aims at exploiting the very fast emission of prompt photons due to effects such as Cherenkov radiation or cross-luminescence [42–46]. Most commonly, these mechanisms are not used in PET systems, as the readout electronics need to provide a very high bandwidth for these very fast signals. However, in recent years, the use of Cherenkov light for fast TOF-PET has been discussed intensively and validated with bench-top experiments [43, 47–50]. For the example of Cherenkov emission in bismuth germanate, ${\hbox {Bi}}_4{\hbox {Ge}}_3{\hbox {O}}_{12}$ (BGO), only about 17 photons are emitted per $\gamma$-interaction, which are mixed with the slower scintillation photons from BGO [51]. In order to distinguish these two photon regimes, the bandwidth of the readout electronics needs to be high, similar to the case of very fast signals. Most importantly, fast timing in single-photon detection is mainly connected to a high SPTR and a good SNR, to which both the photosensor and the electronic readout contribute. For the readout electronics, this means that the discrimination stage and validation scheme need to be carefully designed, keeping in mind that true events and dark counts have the same general shape. Ideally, the precision of any employed time-to-digital converter (TDC) is higher than the SPTR of the SiPM to truly measure the limits of the detector materials and get the best possible timing resolution.

**Table 1:** Authors’ wish list for electronic readout circuits in PET applications

**Requirement**	**Goal**
Form factor and packaging	As small as possible, CSP or BGA, not COB
Power consumption	$\le$ 10 mW per channel including analog and digital part
Data rate requirements	Aim for Mcps per channel to enable single-photon trigger regimes
MR compatibility	Yes
Time-of-flight resolution	$\le$ 100 ps (FWHM) for clinical PET detectors
Single-photon time resolution	$\le$ 25 ps (FWHM) for state-of-the-art SiPMs

### Requirements regarding time-of-flight performance

The TOF resolution or CTR in PET systems generally depends on all components of the readout chain (scintillator, SiPM and readout) employed [18]. Currently, a state-of-the-art TOF performance of 178 ps is achieved with the Biograph Vision scanner fabricated Siemens Healthineers on system level [8]. On benchtop level, discrete electronics read out via oscilloscopes provide performance benchmarks for advanced scintillation materials and novel SiPM technologies. In coincidence experiments with two 3$\times$3$\times$19 mm^3^ LYSO:Ce,Ca crystals and Broadcom NUV-MT SiPMs (3.8$\times$3.8 mm^2^), a coincidence time resolution (CTR) of 95 ps (FWHM) has been measured [52].

The fundamental time resolution of a scintillation process with a certain scintillation rise time $\tau _r$, decay time $\tau _d$ and ILY is defined by photostatistics. In addition to the scintillation emission, the light transfer efficiency (LTE) in the crystal and the photon detection efficiency (PDE) has to be considered. Corresponding parameters are the SPTR of the SiPM and the photon time transfer spread (PTS), which includes firstly the scintillation propagation in the crystal and secondly the travel path of the 511 keV $\gamma$-photons. Combining these parameters, an equation for the lower bound of the time resolution achievable with a scintillator-based detector can be given [18, 53]:

2 $$\begin{aligned} \textsf{CTR} = 3.33 \cdot \sqrt{\frac{ \tau _\textsf{d,eff} \cdot (1.57 \tau _\textsf{r} + 1.13 \cdot \sigma _\mathsf {SPTR+PTS})}{\textsf{PDE} \cdot \textsf{ILY}_\mathsf {@energy} \cdot \textsf{LTE}}} \end{aligned}$$

It should be noted that this equation does not include the emission of prompt photons, e.g., Cherenkov emission in BGO as discussed in Section "Prompt photon emission/Single-photon detection". However, Eq. 2 was proven to be extremely predictive considering scintillators with negligible prompt photon emission [47].

**Fig. 3:**
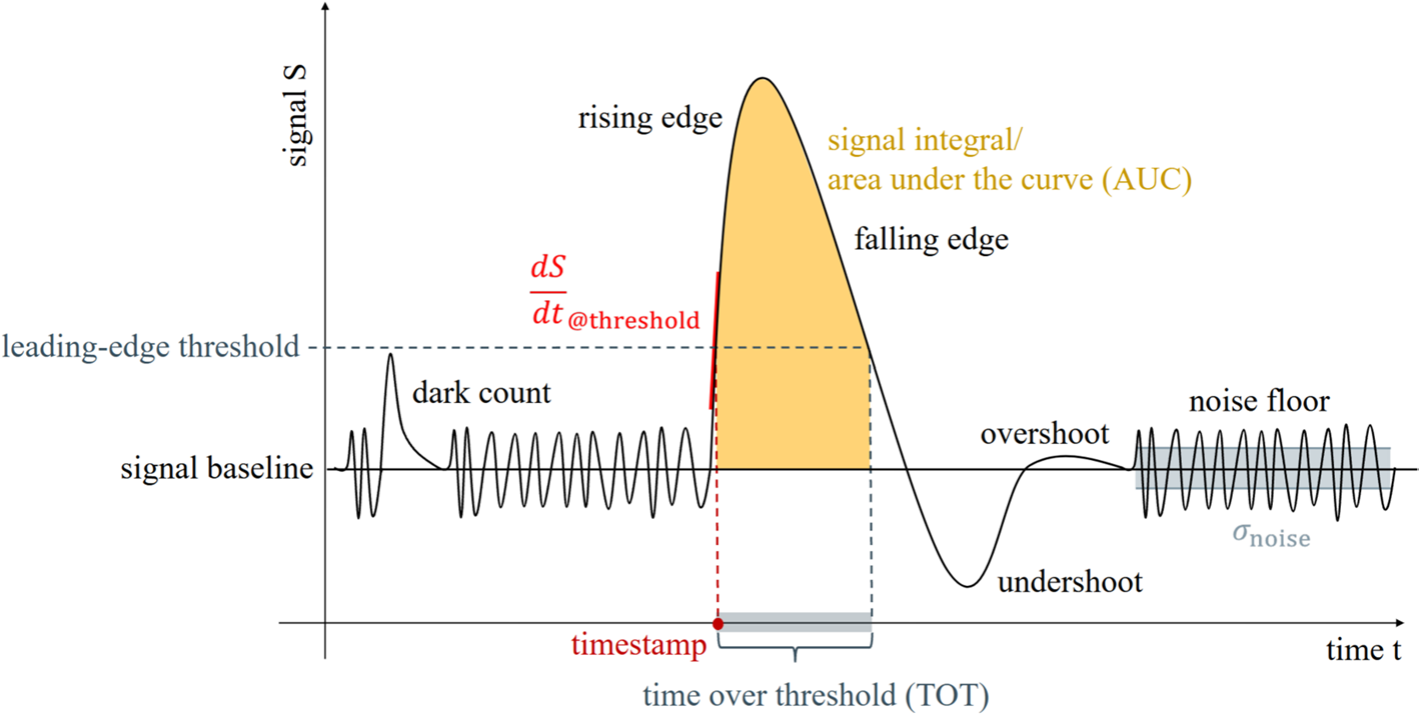
Illustration of the characteristics of a typical analog electronic signal in PET, with the usual parameters to be digitized

Regarding the readout electronics, the most important effect limiting the time resolution is the signal slope at a given leading-edge threshold and the electronic noise $\sigma _\textsf{noise}$ (c.f. Section "Signal characteristics" and Fig. 3). Given a defined leading-edge threshold, the electronic noise can transfer a time jitter in the threshold-signal crossing time, which influences the time resolution and can be described by the fraction [18, 41, 54]

3 $$\begin{aligned} \sigma _\textsf{t} = \frac{\sigma _\textsf{noise}}{\frac{\textsf{dS}}{\textsf{dt}}_\mathsf {@threshold}}. \end{aligned}$$

The signal slope $\frac{\textsf{dS}}{\textsf{dt}}$ increases with increasing number of detected photons (considering a short time period smaller the SPAD signal rise time). Therefore, the electronic noise plays an inferior role in PET measurements, which exploit scintillation emission in contrast to SPTR or multi-photon coincidence time resolution (MPCTR) measurements (c.f. Section "Characterization protocol"). The SPTR or MPCTR obey purely statistical influences. The term $\sigma _\textsf{t}$ is not correlated to these terms in Eq. 2 and adds an additional contribution on the CTR. It should further be noted that the signal slope $\frac{\textsf{dS}}{\textsf{dt}}$ is also influenced by the bandwidth $\textsf{BW}$ with the slope being proportional to the bandwidth and antiproportional to the time jitter:

4 $$\begin{aligned}&\frac{\textsf{dS}}{\textsf{dt}}_\mathsf {@threshold} \propto \textsf{BW} \end{aligned}$$

5 $$\begin{aligned}\sigma _\textsf{noise} \propto \sqrt{\textsf{BW}} \end{aligned}$$

6 $$\begin{aligned}&\sigma _\textsf{t} \propto \frac{1}{\sqrt{\textsf{BW}}} \end{aligned}$$

It is important to note that Eqs. 4 and 6 are only valid when the bandwidth of the electronics is limiting the slew rate of the signal. It can be shown that the optimal bandwidth of the preamplifier is the one needed to match and preserve the slew rate of the input signal [63].

### In summary: What do we need?

Summarizing the requirements stated above, the authors have collected a “wish list” in Table 1 to quantify the requirements posed towards a readout and digitization circuits for PET applications. The values stated for specific performance parameters aim to push the current limits in the field.

## Signal characteristics and digitization

### Signal characteristics

Generally, a PET detector’s signal is the amplitude response to an incident $\gamma$-photon, i.e., a registered voltage or current, over time. More details on the signal generation can be found in Section "Detector-specific requirements". The signal is characterized by first its rising and then its falling edge for a positive polarity or vice-versa if the polarity is negative. Figure 3 illustrates the signal shape for a signal with positive polarity, meaning the amplitude of the signal is registered with a positive sign. The signal rises from its baseline and decays with multi-component exponential character, whereby it is possible that it undershoots or overshoots the baseline before baseline recovery depending on the employed readout electronics. However, if longer than the expected time difference between two events, over- and undershooting disrupts a stable baseline and a well-defined leading edge threshold. Therefore, the design of the readout electronics should aim for fast decay and quick baseline recovery as sketched in Fig. 3. Signal over- and undershoots may even be implemented intentionally for bipolar shaping or encoding time and energy information in a combined signal [55]. The rise time of the signal, i.e., the steepness of the rising edge ($\frac{\textsf{dS}}{\textsf{dt}}$), can vary with the amount of energy deposited. This variation causes a time walk effect, i.e., a jitter in the generated timestamp depending on the signal energy and amplitude. Additionally, the path lengths of the electronic readout channels can contribute to time skews on the temporal information of the signal that need to be corrected via dedicated calibration procedures [56, 57]. The signal baseline is subject to small variations due to electronic noise, whose average width is labeled with $\sigma _\textsf{noise}$ (c.f. Eq. 3).

**Fig. 4:**
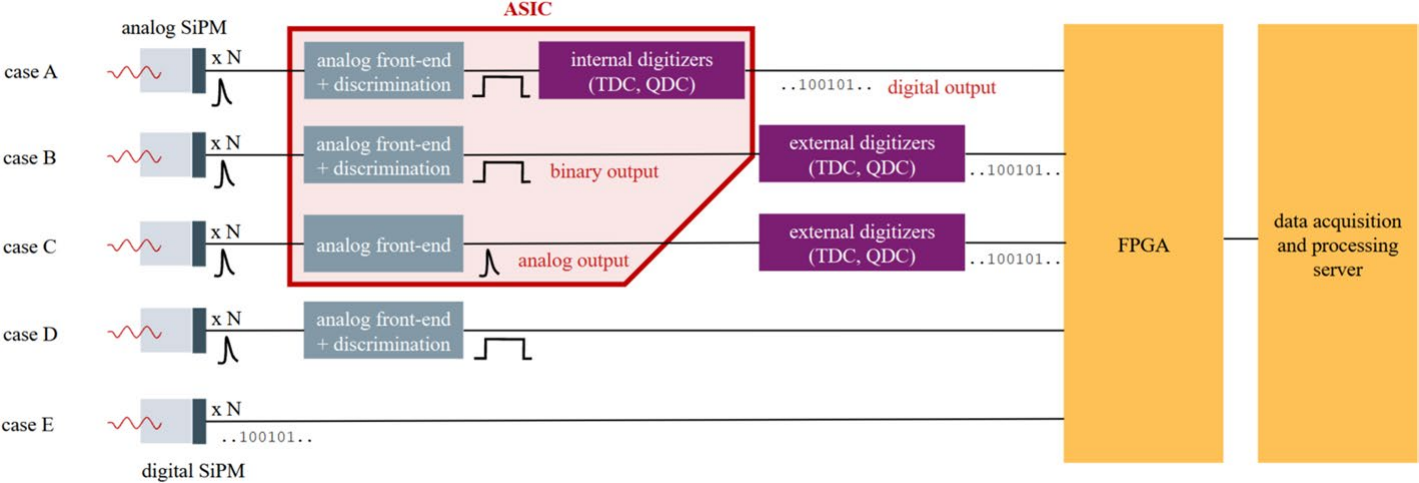
Sketch of different readout schemes employing an ASIC (cases A, B and C) or omitting it (cases D and E). The output of an ASIC can be analog, binary (discriminated pulse) or digital. This review focuses on the schemes with ASICs as depicted in A, B and C

### Signal digitization by electronic circuits

Assuming that the signal sketched in Fig. 3 stems from an SiPM in a PET detector block, the readout electronics typically amplify the signal before triggering on them. The signal can be read in voltage or current mode. In voltage mode, the SiPM current is forced on a known impedance. The voltage drop over this impedance is read as a signal. In current mode, the current is either read via a virtual ground (negative feedback) or a current conveyor (positive feedback) [58–60]. Specifically for the negative feedback current mode, the circuit is prone to instability issues and largely influenced by the capacitance of the employed SiPM. Depending on the readout mode (voltage or current readout), amplification is achieved using transimpedance amplifiers, common source stages with resistive or capacitive feedback, common gate stages or current conveyors and in rare cases operational amplifiers. Among these, transimpedance amplifiers hold the advantage of a faster signal, but are prone to oscillatory behavior. Operational amplifiers might be used as shapers, but limit performance with their noise behavior and bandwidth. The trigger is usually implemented using a discriminator or comparator, which outputs a rectangular pulse once the signal surpasses an amplitude threshold relative to the baseline. The rectangular pulse is fed into a TDC for digitization, where the rising edge of the discriminated pulse is used as a timestamp estimate. As a measure of the energy of a signal on the other hand, integration of the signal charge by a charge-to-digital converter (QDC) or determination of the width of the pulse by a time-over-threshold (TOT) method are used (see Fig. 3). As an example among many other architectures, the digitization can be realized by employing ring oscillators and determining the phase difference (e.g. Vernier architecture), charging an array of capacitors or counter-based architectures [61–63]. The part of the readout electronics up to the digitizers is typically referred to as electronic front end. In recent years, high-frequency (HF) electronics have shown outstanding potential as front ends for TOF-PET applications [41, 54, 64, 65]. The front end with or without the digitizers can be incorporated into a so-called application-specific integrated circuit (ASIC), which is a highly integrated electronic circuit with a small form factor produced as a standalone electronic part or chip in a complementary metal-oxidesemiconductor (CMOS) process housing the readout electronics for dozens of PET detector channels. Not all ASICs implement all required components in order to get to the final digitized information (c.f. Fig. 4). There are purely analog ASICs that might only implement analog signal amplification leading to an analog output or additional leading-edge discrimination that does not directly output digital information, but a discriminated pulse (binary output, c.f. Fig. 4). As a results, this requires further external components like a TDC. The ASIC communicates the analog information to these external digitizers or already digital information typically to a field-programmable gate array (FPGA), collecting and transporting the data to the acquisition system.

With the individual SPADs in an SiPM being prone to thermal breakdowns and hence dark counts, noise rejection schemes are key to distinguish true $\gamma$-events from noise. Noise rejection schemes at an early stage should prevent the ASIC data rate from being saturated and allow to trigger dead-time prone readout functions only for the digitization of true $\gamma$-events. The simplest form of noise rejection is the configuration of a sufficiently high threshold on the rising edge of a true signal that will not allow to trigger on individual SPAD triggers, as depicted in Fig. 3. Since a higher threshold can result in deteriorated timing performance due to worse photostatistics and might be unsuitable for single-photon trigger schemes, popular circuits employ a multi-threshold trigger logic for noise rejection [22]. Trigger groups or logic interconnections are possible to reject noise as well and are common measures to reduce the number of required readout channels.

PET relevant data processing steps, like calculations on single events (singles) and coincidence processing, might be implemented in further FPGA-based electronics components before data is sent towards a computer. The specific processing steps in the data pipeline are dependent on the setup. In larger setups or medical scanners, typically more processing is performed in the system, whereas in benchtop experiments access to the raw ASIC data output is often preferred and allows for more advanced processing techniques interesting for research (see Fig. 4) [66]. There are two main challenges for the task of registering $\gamma$-interactions with high sensitivity and precision. First, it is difficult to differentiate between true signals and noise due to a high dark count rate for most SiPM technologies. Furthermore, a generally high data rate due to a high activity in the FOV and thus high interaction rates in the scintillator causes a high rate of SiPM pulses, which is demanding.

**Fig. 5:**
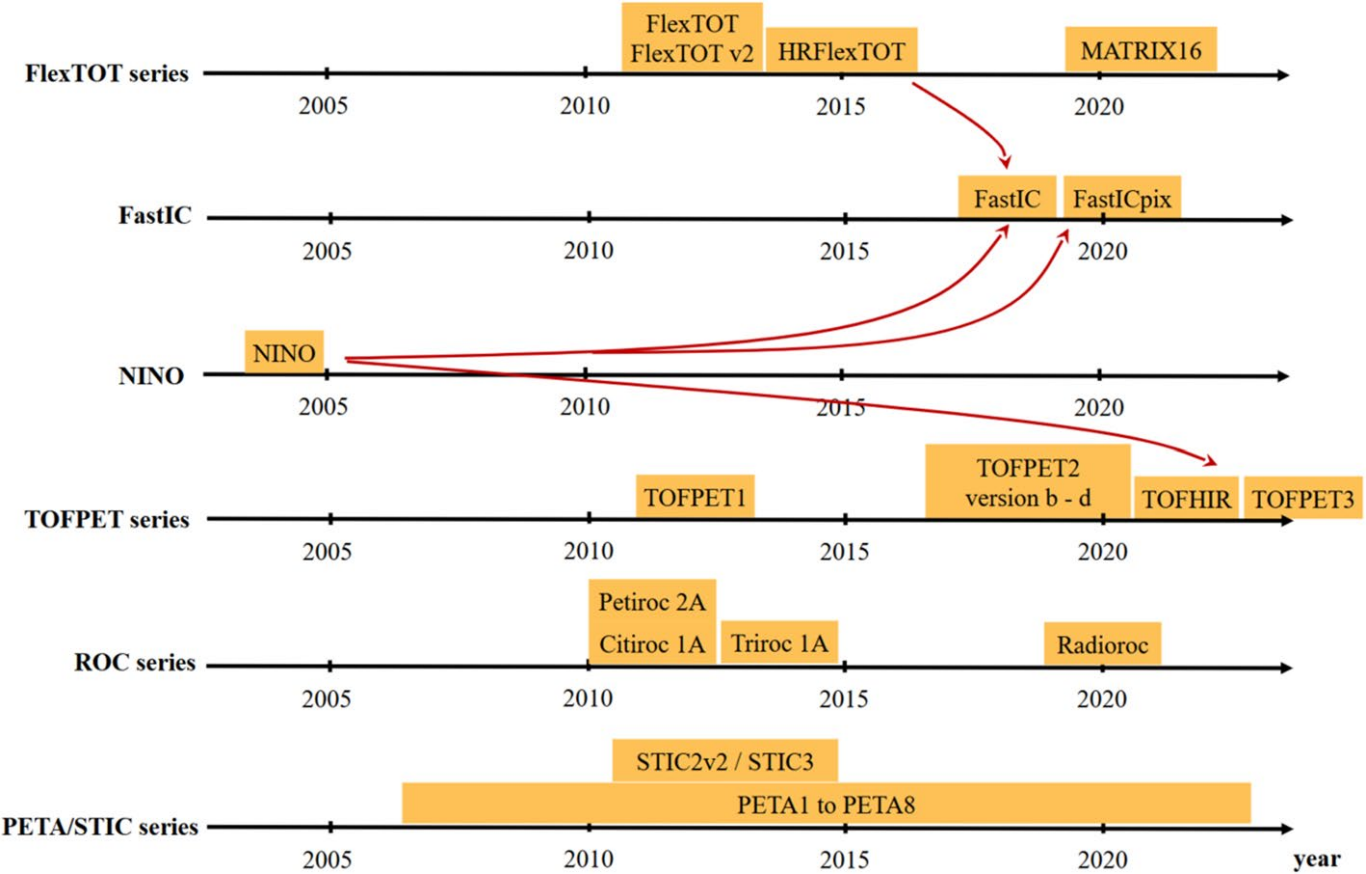
Timeline of the publication dates of the larger ASIC series. Delays are possible with respect to the true year of release due to later publication of characterization results. The influence of the NINO and HRFlexToT ASICs on other designs is indicated by arrows. Graphic taken from [67] and modified

### Flexibe readout architectures omitting the ASIC

ASICs have the advantage to be highly integrated, offering the possibility to readout several dozen channels with a small form factor. Due to their compactness, they have become unavoidable. However, the production of ASICs is time consuming and costly when prototyping starts, and only become more cost-effective when produced in large batches. Hence, there has been several investigations on how to omit the ASIC in order to make the PET architecture more flexible and cost-effective to test new ideas [50, 68–70]. There are two main ways of omitting the ASIC, a) discrete circuit design including TDCs followed by data handling in FPGAs, and, b) direct waveform digitization in FPGAs or analog digitizers, including online or offline data analysis. Due to the use of discrete and commercially available components, development periods are fast and different readout architectures can be tested. Once the digitization path is set-up, e.g., via low voltage differential signaling (LVDS) and external TDCs and QDCs, changes in the front end can be implemented flexibly and optimized for different detector materials. Although the scaling of such research prototypes to whole PET systems is difficult, they give valuable insight in readout strategies and can further guide the development of ASICs.

A rather new approach of digitizing the SiPM signals is to directly route the signals in the FPGA, e.g., single-photon signals from Cherenkov emission in monolithic BGO detectors using SiPM arrays [68]. Readout was done by a high-frequency circuit followed by leading-edge discrimination, as also implemented in [71]. Due to the low ILY of BGO and the low photon density on the multiple SiPM channels, the resulting LVDS signals of a single channel is very similar to a digital data-stream [69]. This is received by fast data-links on the FPGA and digitized. Depending on the segmentation on the SiPM array this readout is also applicable for lutetium-based scintillators [70].

A second approach makes use of the digital nature of SPAD signals and discriminates the SPAD signals directly on the SiPM, with the SPAD itself delivering discrete pulses with the rising edge corresponding to the optical photon detection time. The digitization can be done by 3D integration of the SiPM and readout electronics or in a monolithic approach sacrificing some active area [72–75].

Depending on its architecture, the implementation of the digital SiPM is not expected to produce higher amounts of data than comparable analog solutions [26, 27, 40], the requirements posed to the FPGA might be more elaborate for other approaches [74]. In the digital approach, a compromise has to be made between retrieved information and trigger or readout limits regarding the data rate. If available, the full information of the data can be used to implement sophisticated estimators, e.g., $\gamma$-interaction reconstruction or maximum likelihood time estimations are possible [56, 76, 77].

Apart from the digital SiPM [73], which has been implemented in a clinical PET system [78], the solutions introduced in this section are directed towards research prototypes with the aim to optimize performance and are currently not used in clinical PET machines. Therefore, this review will focus solely on ASIC implementations and scalable, system-applicable electronics.

**Fig. 6:**
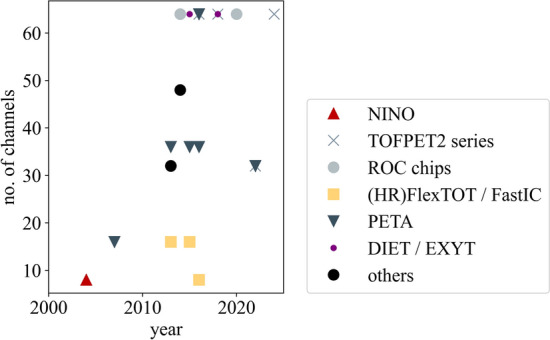
Evolution of the number of ASIC readout channels per chip for PET applications over the past twenty years. Delays are possible with respect to the true year of release due to later publication of characterization results. Data have been compiled using the references in Table 2

**Fig. 7:**
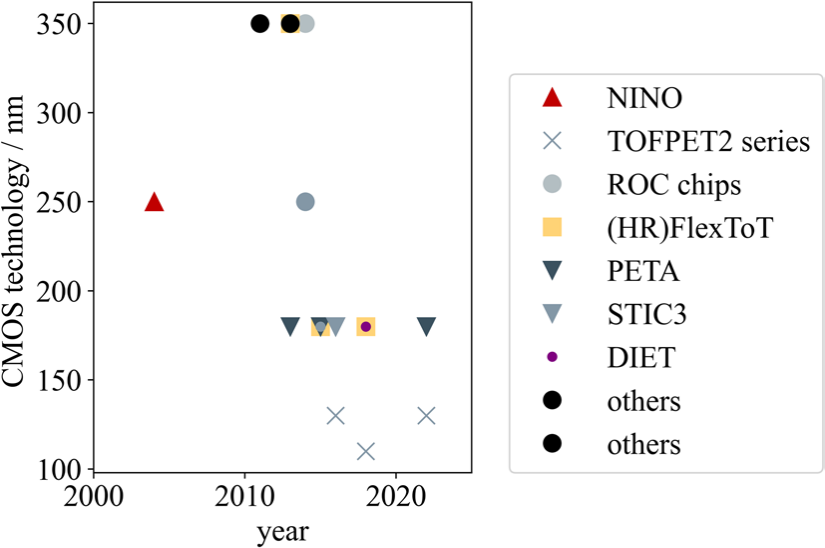
Evolution of manufacturing process of ASICs for PET applications over the past twenty years. Delays are possible with respect to the true year of release due to later publication of characterization results. Data have been compiled using the references in Table 2

**Fig. 8:**
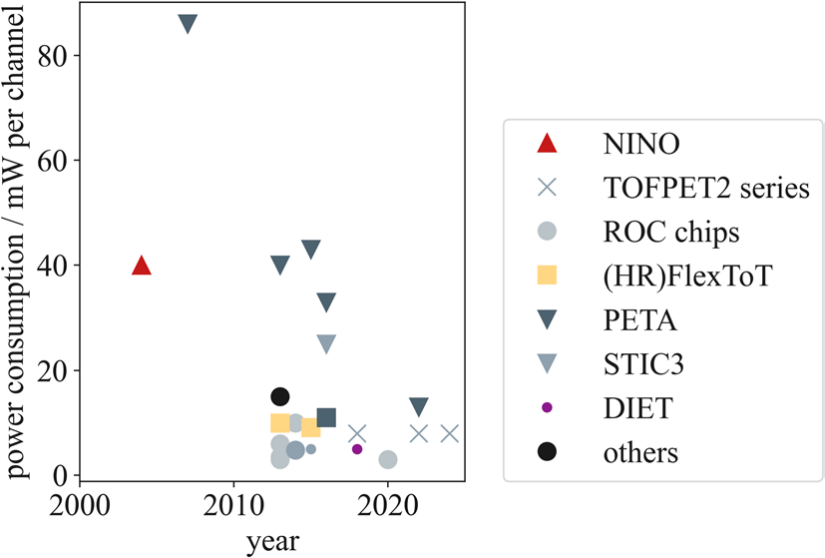
Evolution of the ASIC power consumption per channel for PET applications over the past twenty years. The reported power consumption mixes front-end only and full architecture (digtal and analog part) values reported, depending on their availability in publications. Delays are possible with respect to the true year of release due to later publication of characterization results. Data have been compiled using the references in Table 2

## ASICs in PET

This section focuses on ASICs which are specifically applied to detector readout in PET applications. Other use cases are not described to keep within the scope of this review article. Additionally, only commercially available ASICs and research prototypes are taken into consideration as information on industrial solutions is usually subject to non-disclosure and, hence, barely available. While some ASICs have been specifically designed for PET, other have found their way to PET via high-energy physics (HEP) and space applications using similar solid-state detectors. An overview on the release dates of the larger ASIC series in PET is provided in Fig. 5. This study intends to provide an overview over the many ASIC designs and characteristics (c.f. Tables 2 and 3 as well as Figs. 6, 7 and 8), each designed for a specific application and purpose in PET, without selecting one ASIC among many or depicting one circuit design as more mature or better than others.

**Table 2:** ASICs in PET: overview on the design specifications of different ASICs designed for high energy physics (HEP) and PET applications. Tabular taken from [67] and modified

ASIC	Developer	Year	Package size ${\textbf {mm}}^2$	No. of channels	CMOS techn./nm	References
TOFPET1	PETsys	2012		64	130	[79]
TOFPET2b/c	PETsys	2016	14.0 $\times$ 14.0	64	110	[22, 80]
TOFHiR 2A	PETsys/CERN	2022	8.5 $\times$ 5.2	32	130	[81]
TOFPET3	PETsys	2024		64		[82]
Citiroc 1A	Weeroc			32		[83, 84]
Petiroc	Weeroc	2013		16		[83, 84]
Petiroc 2A	Weeroc			32		[83]
Triroc 1A	Weeroc	2014		64	350	[85]
Radioroc	Weeroc	2020		64		[25]
NINO	CERN	2004	2 $\times$ 4	8	250	[86, 87]
FlexToT	ICCUB/CIEMAT	2012	9.0 $\times$ 9.0	16	350	[88–91]
HRFlexToT	ICCUB/CIEMAT	2018		16	180	[24, 87, 92]
MATRIX16	ICCUB/CIEMAT	2021	4.57	16	180	[93]
FastIC	CERN/ICCUB	2016		8	65	
FastICpix	CERN/ICCUB	2022				[94, 95]
PETA4	ZITI	2013	5.0 $\times$ 5.0	36	180	[23]
PETA5	ZITI	2015	5.0 $\times$ 5.0	36	180	[96]
PETA6	ZITI	2016		36	180	[97, 98]
PETA8	ZITI	2022	5.0 $\times$ 5.0	32	180	[98, 99]
PETAT1	ZITI	2022	5.0 $\times$ 6.0	32	180	[100]
STIC3	ZITI	2014		64	180	[101]
EXYT	Tsingua University	2015		64	180	[102]
DIET	Tsingua University	2018	3.0 $\times$ 3.0	64	180	[103]
TODPET	University of Tokyo	2014		48	250	[104]
MPPC32	JAXA	2013		42	350	[105]
IMOTEPAD	IPHC	2011		64	350	[106]

**Table 3:** ASICs in PET: overview on the hardware characteristics and required supply voltages of different ASICs designed for HEP and PET applications. Tabular taken from [67] and modified

ASIC	Supply voltage/V	Power consumption/mW/ch	Signal polarity	Input stage mode	Charge measurement	Output	References
TOFPET1		8 to 11	pos	Current	TOT	Digital	[79]
TOFPET2b/c	1.2	5 to 8	pos	Current	QDC, TOT, mixed	Digital	[22, 80]
TOFHiR 2A	1.2						[81]
TOFPET3		8	pos, neg	Current	QDC, TOT, mixed	Digital	[82]
Citiroc 1A	3.3	3 (excl. buff.)	pos	Current	QDC		[83, 84]
Petiroc	3.3	3.5 (excl. buff.)		Current	TOT		[83, 84]
Petiroc 2A	3.3	6	pos, neg	Current	TOT		[83]
Triroc 1A	3.3	10 (excl. buff.)	pos, neg	Current	QDC	Digital	[85]
Radioroc		3 (excl. TDC)		Current	TOT		[25]
NINO		27 to 40	diff	Voltage	TOT	Analog	[86, 87]
FlexToT	pos	10		Current	QDC	Analog	[88–91]
HRFlexToT		3.5	pos	Current	QDC	Analog	[24, 87, 92]
MATRIX16	1.8	9.1	pos	Current	QDC	Digital	[93]
FastIC	1.2	12	pos, neg, diff	Mixed	QDC	Analog	
FastICpix							[94, 95]
PETA4	1.8	40	neg, diff	Mixed	QDC	Digital	[23]
PETA5	1.8	43	neg, diff	Mixed	QDC	Digital	[96]
PETA6	1.8	33	pos, neg, diff	Mixed	QDC	Digital	[97, 98]
PETA8		13			QDC	Digital	[98, 99]
PETAT1		13			QDC	Digital	[100]
STIC3		25	diff	Voltage	tot	Digital	[101]
EXYT		< 5	diff, pos, neg	Mixed			[102]
DIET		5	diff, pos, neg	Mixed	QDC		[103]
TODPET		4.8	diff, pos, neg	Mixed	TOT		[104]
MPPC32		15			TOT		[105]
IMOTEPAD	3.3	16.8		Mixed	TOT	Digital	[106]

### ASIC families

In many cases, developments in HEP applications also find their way into the field of PET. One example is the **NINO** ASIC released in 2004, which was originally developed for TOF detectors in HEP applications, particularly the ALICE experiment at European Council for Nuclear Research (Conseil Européen pour la Recherche Nucléaire) (CERN), Meyrin, Switzerland [86, 87, 94].

Institutions developing ASIC often design and produce whole families of ASICs, while the chips undergo several iterations. One of these families started in 2012, when Institut de Ciènces del Cosmos at University of Barcelona (ICCUB), Spain, and Centre for Energy, Environmental and Technological Research (Centro de Investigaciones Energéticas, Medioambientales y Tecnológicas) (CIEMAT), Madrid, Spain released the **FlexTOT** ASIC. Six years after the release of the FlexTOT, i.e., in 2018, ICCUB and CIEMAT published the high-resolution FlexTOT (HRFlexTOT) ASIC. In 2021, the MATRIX16 was released to provide digitizers to the outputs of the HRFlexTOT [24, 87–93].

In 2016, CERN, Meyrin, Switzerland, and ICCUB, Spain, jointly released the **FastIC** ASIC based on the architecture of the HRFlexToT ASIC for HEP and other applications such as light detection and ranging (LiDAR) or PET [94, 95, 107, 108]. The FastICpix design has been developed within an ATTRACT Phase I grant [109] and is currently studied in simulations. It involves sensor segmentation to reduce the effective capacitance and its impact on the timing resolution, similar to the DFG-SNF Digilog project [72].

From 2007 to 2017, the Institute of Computer Engineering (ZITI) at Ruprecht-Karls University Heidelberg, Germany, developed a series of ASICs called **PETA** (Position Energy Timing ASIC) [23, 96–101, 110–115]. Since differential readout limits the sensitivity to pick up, PETA prototypes have been evaluated as candidates for hybrid PET/magnetic-resonance (MR) systems, e.g., in projects such as HYPERImage and Sublima (PETA2 and PETA3) [116] or the SAFIR project at ETH Zurich, Switzerland (PETA6SE, PETA8) [33, 117]. In 2014, for the EndoTOF-US project, the ZITI at Ruprecht-Karls University Heidelberg, Germany, released the STIC2v2 ASIC (Silicon Photomultiplier Timing Chip).

The Weeroc ASIC family, or **ROC series**, is a considerably large ASIC family, which has been commercialized by a spin off of the Omega microelectronics group at IN2P3/CNRS, Paris, France. ASICs at Weeroc have been designed for various applications, comprising HEP, space, homeland security and medical imaging [30, 83, 84, 118]. This review only focuses on the chips applicable for PET imaging and SiPM readout, for example Petiroc. In 2014, for the EU project TRIMAGE, Weeroc released the Triroc 1A to be integrated into a hybrid PET/MRI/electroencephalogram (EEG) system. While originally developed for the detection of cosmic rays, $\gamma$-rays and muons in space applications in 2020, Radioroc has recently been considered a suitable ASIC for PET applications [25].

As a first exemplar of the TOFPET ASIC series, the 64-channel TOFPET1 ASIC was released by PETsys Electronics S.A., Lisbon, Portugal in 2012. As a competitor to the STIC ASIC, it was intended as PET electronics within the EndoTOFPET-US project. Five years after, in 2017, the successor of the TOFPET1 ASIC, the TOFPET2 ASIC was released by PETsys Electronics S.A. First published in 2022, the TOFHiR 2A ASIC was presented, which was originally designed for the detection of events in the compact myon solenoid (CMS) Barrel Timing Layer at CERN [22, 26, 40, 79–81, 119–121]. In 2024, PETsys Electronics S.A. officially presented their most recent prototype of the TOFPET ASIC series, the TOFPET3 ASIC [82].

In 2015, Tsingua University, China, published the EXYT ASIC. Three years later, in 2018, they released the DIET ASIC [102, 103, 122].

Some institutions developed further ASICs for PET applications that cannot be assigned to any of the larger ASIC families. In 2014, the University of Tokyo, Japan, released the TODPET ASIC [104]. In 2013, the MPPC32 ASIC has been published by JAXA [105]. The IPHC, Strasbourg, France, released the 64-channel IMMOTEPAD ASIC as dedicated electronics for micro-channel plate (MCP) readout in small-animal PET systems [106].

The following paragraphs elaborate on the hardware characteristics and performance of the ASICs currently available for PET applications. Furthermore, we aim to put them into context with the aforementioned requirements (c.f. Section "Requirements for scalable, system‑applicable readout electronics"). For better readability we refrain from incorporating the references for the respective ASIC, which have been cited in Section "ASIC families".

### Form factor and packaging

ASICs currently available for PET applications feature between eight and 64 readout channels with a package size of up to few square centimeters, keeping a reasonable size for integration on system electronics (c.f. Table 2. While the NINO ASIC and FastIC started off with eight readout channels and the earliest Petiroc version and the FlexToT and HRFlexToT were designed for 16 channels, later ASIC versions were capable of reading 32 to 36 channels (TOFHiR 2A, Citiroc 1A, Petiroc 2A, PETA4 to PETA8, PETAT1, MPPC32) or even 64 channels (TOFPET1, TOFPET2, Radioroc, Triroc 1A, STIC3, DIET, EXYT). The trend towards an increased number of readout channels can also be seen in Fig. 6. The CMOS technology, in which the ASICs are produced, has seen a trend from larger to smaller form factors, facilitating the incorporation of more readout channels. Earlier ASIC versions started with 350 nm CMOS technology (Triroc 1A, FlexToT, MPPC32, IMOTEPAD), which decreased via 250 nm (NINO, TODPET) to 180 nm and below (TOFPET sereis, HRFlexToT, PETA series, EXYT, DIET). More details can be found in Table 2 and Fig. 7.

It is striking that while the number of readout channels and CMOS technology are more or less consistently reported for all investigated ASICs, the actual chip size often remains unmentioned in publications, making it difficult to identify the space requirements for the system integration of particular ASICs.

### Event digitization

Required to digitize signal pulses from PET detectors (c.f. Section "Signal characteristics"), ASICs vary in their input stage design and branching to tackle this complex task. ASIC designers may have developed highly flexible ASICs with adjustable input stage parameters, trigger logic and power consumption or may have tailored their ASIC precisely to its task, only allowing to read a specific signal polarity. Furthermore, there are ASIC solutions with integrated digitizers (QDC, TDC) available (TOFPET series, PETA series), while other rely on external TDCs (Radioroc, NINO, HRFlexTOT), e.g., an oscilloscope or CAEN modules. Additionally, the applied method to access a signal’s energy may vary, since some ASICs offer a TOT method (NINO, TOFPET1, Petiroc, Radioroc, FastIC, HRFlexTOT, TODPET, MPPC32, IMOTEPAD), while others integrate the signal charge (TOFPET2, PETA series, Triroc 1A, DIET). This information is summarized in Table 3.

The following paragraphs briefly explain the various amplification and trigger schemes applied depending on the ASIC used.

The NINO ASIC provides differential inputs for up to eight channels. The energy of a signal can be assessed via a TOT method. The output of the NINO ASIC is purely analog, i.e., it requires external TDCs for digitization.

For the FlexTOT, a low input stage impedance of 30 $\Omega$ is realized by the use of two feedback loops. In each channel, the input signal is divided and amplified in three different stages. One of these stages either triggers on channel-individual signals or reads all channels by a common $\mathsf {fast-OR}$ trigger. A TOT measurement can be performed on a discriminated pulse, which is output by the second stage. For the FlexTOT v2, the TOT linearity has been extended from 2.5 mA to 18 mA to 0.7 mA to 18 mA. The remaining third stage is able to classify pile-up events. Both the FlexTOT and its successor the HRFlexTOT feature analog outputs. With the release of MATRIX1, a ring oscillator was implemented to process events in clusters of four of the 16 channels, resulting in a time jitter of less than 8 ps (RMS).

The FastIC features eight channels with an intrinsic gain of $10^5$ to $10^6$, capable of handling positive, negative and differential signals in current mode. The design of the FastIC ASIC is compatible with the picoTDC designed at CERN [123]. The FastIC replicates the signal into three different branches and amplifies it. In the first branch, the signal is highly amplified with a low-threshold current discriminator to generate a timestamp on single-photon trigger level per channel or a $\mathsf {fast-OR}$ trigger considering the output of all channels as for the HRFlexTOT. The second branch applies a higher trigger to a signal with lower amplification, allowing for a second trigger that can either be an $\textsf{OR}$ between all eight channels or a cluster trigger on the $\textsf{SUM}$ ($\Sigma$) of all channels. The third branch employs a peak detector and hold (PDH) and a discriminator for a TOT measurement to assess the signal charge. The FastICpix employs a TDC based on a 2 GHz ring oscillator to achieve a time resolution of 10 ps, which is in line with the “10 ps challenge” in PET [9, 124–126].

PETA ASICs feature a mixed-mode input stage, which allows for single-ended or differential readout, with a low input stage impedance between 7 $\Omega$ and 10 $\Omega$, depending on the circuit version. PETA1 to PETA5 were designed to only read signals with negative polarity, while PETA6 allows to read positive as well as negative signal polarity. PETA ASICs digitize the timestamp and energy of a signal in two different branches, where a charge integration method is employed. PETAT1 was designed to allow communication between chips, possibly allowing to omit an FPGA per chip [100]. The STIC3 uses a TOT method to assess the signal energy and is designed for single-ended SiPM readout.

Among the ROC series, Citiroc 1A reads signals with positive signal polarity. The charge output is multiplexed and the signal energy is digitized using a charge integration method, which is linear up to 2500 p.e. The trigger, upon which the timestamp is evaluated, is a logical $\textsf{OR}$. Petiroc 2A supports signals with positive and negative polarity. Similar to Citiroc 1A, Petiroc 2A uses an $\textsf{OR}$ trigger for the timestamp generation, but measures the signal energy via a TOT method, whereby the TDC and analog-to-digital converter (ADC) are integrated into the ASIC. Triroc 1A supports positive and negative signal polarity. Similar to Citiroc 1A, Triroc 1A uses an $\textsf{OR}$ trigger for the timestamp generation and assesses the charge via an integration method, whereby the TDC and ADC are integrated into the ASIC. Radioroc operates in current-mode and requires an external TDC. The signal is duplicated into three branches and amplified, where the first branch uses a preamplifier with an adjustable gain and two discriminator to generate a timestamp. This branch is also used to measure the signal’s energy via a TOT method linear up to 2000 p.e. The second and third branch employ a high-gain and a low-gain amplifier, respectively, which allow to separate single p.e. triggers and enable to reject triggers with respect to the deposited energy.

The TOFPET series provides ASICs that allow for high flexiblity in terms of configuration. For TOFPET1, the configuration of the input stage impedance between 10 $\Omega$ to 60 $\Omega$ optimizes the timing performance of the circuit. Following the input stage, two different branches are used to generate a timestamp and to measure the deposited energy via a non-linear TOT method. TOFPET2 still allows to configure the input stage impedance. After the input stage, the signal is duplicated into two branches, one assigning a timestamp, the other one measuring the energy of the signal via a charge integration method. The timing branch employs a two-threshold trigger logic to reject dark counts without dead time, while the energy branch employs a third discriminator for energy validation. The trigger logic can be re-configured using any pair of discriminators in a single-, double- or three-threshold trigger logic.

The EXYT ASIC employs fast timing outputs and common resistive networks to assess the signal energy and to position events. It can read positive, negative and differential signals. While the fast timing channel features an input impedance of 46 $\Omega$, this parameter is reduced to 34 $\Omega$ for the energy and positioning channel.

The IMMOTEPAD ASIC executes the energy measurement within the analog part of the ASIC. The digital part features a delay-locked loop (DLL) and a TDC to generate a timestamp.

As all these ASICs vary in their design, it is difficult and maybe even inappropriate to consistently report their digitization schemes. However, the exact definition of the ASIC output and easy accessibility of the output protocol are key parameters to enable system integration.

### Power consumption

As discussed in Section "Power consumption", the power consumption of an ASIC is essential to decide on the system integrability of an ASIC, with the current consumption becoming especially important for hybrid systems. The power consumption of ASICs for PET application has seen a reduction over the passed decades as depicted in Fig. 8, which is beneficial for system integration. However, the reports are to be treated with caution as it becomes apparent that the power consumption is reported inconsistently among different studies, sometimes including the consumption of the digitizers or FPGAs (TOFPET2, PETA series), sometimes not including these (HRFlexToT, NINO, ROC series). The required supply voltage is sometimes unavailable in reports and the current consumption to our knowledge barely reported in literature, making it a challenging task to identify suitable ASICs for PET/MR applications.

To show the difficulty of comparing the power consumption of ASICs for PET applications, the following paragraphs summarize the power consumption of various ASICs as they are reported in literature.

Characterization studies of the NINO ASIC show a low timing jitter, but report a high power consumption of initially 40 mW per channel in 2004. This was improved to 27 mW per channel in a follow-up version of the chip in 2017.

The power consumption of this circuit is 10 mW per channel (FlexTOT) to 11 mW per channel (FlexTOT v2), which does not include the consumption of an external TDC and FPGA, which are both necessary to digitize the discriminated pulses. For the HRFlexTOT, however, the power consumption was significantly reduced to 3.5 mW per channel, still requiring an external TDC and FPGA, which would consume additional power. The MATRIX16 requires a supply voltage of ${\hbox {V}}_\textsf{DD}$ = 1.8 V, while consuming a power of 9.1 mW per channel in high-power mode. The power consumption can be reduced to 2.9 mW per channel in ultra-low-power mode, at the cost of increasing the time jitter.

The power consumption of the FastIC is in the range of 6 mW to 12 mW per channel using a supply voltage of ${\hbox {V}}_\textsf{DD}$ = 1.2 V, of which about 3 mW are attributed to the input stage of the FastIC.

For the PETA ASICS, the power consumption could be reduced from 86 mW per channel to 33 mW per channel for the analog and up to 8 mW per channel for the digital circuit part from the earliest versions to PETA6. The latest versions, i.e., PETA8 and PETAT1 only consume 13 mW per channel, which reduces the power consumption again by more than 50 % [98–100]. The STIC3 power consumption is lower than the PETA ASIC’s power consumption and amounts to 25 mW per channel.

Citiroc 1A has a power consumption of 3 mW per channel, excluding the ASIC buffers, using a supply voltage of ${\hbox {V}}_\textsf{DD}$=3.3 V. The Petiroc’s power consumption is at a similar level the Citiroc’s with 3.5 mW per channel excluding the ASIC buffers. Compared to its predecessor, the Petiroc 2A’s power consumption was reduced to 6 mW per channel using a supply voltage of ${\hbox {V}}_\textsf{DD}$ = 3.3 V. Triroc 1A power consumption amounts to 10 mW per channel excluding the ASIC buffers using a supply voltage of ${\hbox {V}}_\textsf{DD}$ = 3.3 V. Radioroc consumes 3 mW per channel

Including the integrated TDC, the power consumption of the TOFPET1 ASIC amounts to 7 mW per channel. The TOFPET2 ASIC consumes a power of 8 mW per channel using a 1.2 V power supply, which can be tuned down to about 4 mW per channel at the cost of performance [120]. This includes the power consumption of an FPGA supplied by a 2.5 V line. For the TOFPET3 ASIC a similar value was reported.

The power consumption of the EXYT amounts to less than 5 mW per channel. The DIET ASIC consumes a power of 5 mW per channel.

The TODPET’s power consumption amounts to 4.8 mW per channel, excluding TDCs and ADCs. MPPC32’s power consumption amounts to 15 mW per channel. IMMOTEPAD consumes a power of 16.8 mW per channel, using a supply voltage of ${\hbox {V}}_{\textsf{DD}} =$ 3.3 V.

### Sensitivity and data rate

The achievable data rate or maximum event rate per ASIC channel and also per ASIC is a parameter, which is crucial for system integration, but also very difficult to find as it depends on many factors. The implementation of the readout chain, including FPGA firmware limits, may influence the achievable data rate. Furthermore, when considering reporting sensitivity in terms of coincident $\gamma$-photon detection, the achievable sensitivity strongly depends on the employed detector concept. However, sensitivity is a critical parameter for PET applications as it directly impacts the administered radiation dose and scan time for the patient [2]. Among the ASICs investigated, we were able to identify the following data rate capabilities. The FastIC supports a data rate higher than 50 MHz. The maximum data rate supported for the Triroc 1A is 50 kcps per chip. For TOFPET1, the maximum data rate supported per individual channel is 100 kHz to 160 kHz. TOFPET2 realizes an overall event rate of 600kHz per channel, each channel is multi-buffered by four analog buffers. TOFHIR 2A supports high data output rates up to 7.5 MHz per channel and TOFPET3 supports 500 kHz per channel. As these only refer to about a fourth of all investigated ASICs, this identifies a gap between the hardware characteristics reported and the information needed to reliably identify system-applicable readout electronics.

**Table 4:** ASICs in PET: Performance overview in terms of coincidence time resolution, single-photon time resolution and energy resolution of different ASICs designed for HEP and PET applications. Only results from single detector pixel measurements are displayed

ASIC	SiPM	Crystal	CTR (FWHM) / ps	SPTR (FWHM) / ps	dE/E (FWHM) / %	References
TOFPET2c	BRCM AFBR-S4N33C013	2$\times$2$\times$3 mm^3^ LYSO:Ce,Ca	127	n.a.	10.4	[52]
TOFPET2c	BRCM AFBR-S4N44P014M	2$\times$2$\times$3 mm^3^ LYSO:Ce,Ca	128	n.a.	n.a.	[71]
TOFPET2c	BRCM AFBR-S4N44P014M	3$\times$3$\times$19 mm^3^ LYSO:Ce,Ca	157	n.a.	n.a.	[52]
TOFPET2c	BRCM AFBR-S4N44P014M	2$\times$2$\times$3 mm^3^ BGO	480	n.a.	n.a.	[71]
TOFHiR 2A						
Liroc	FBK NUV-HD-LF M3	n.a.	n.a.	90	n.a.	[25]
Radioroc	BRCM AFBR-S4N44P014M	2$\times$2$\times$3 mm^3^ LYSO:Ce,Ca	83	n.a.	n.a.	[25]
Radioroc	BRCM AFBR-S4N44P014M	3$\times$3$\times$20 mm^3^ LYSO:Ce,Ca	127	n.a.	9.2	[25]
Radioroc	FBK NUV-HD-LF M3	n.a.	n.a.	73	n.a.	[25]
NINO	HPK S13360-3050CS	2$\times$2$\times$5 mm^3^ LSO:Ce,0.4%Ca	93	151	n.a.	[87]
NINO	FBK NUV-HD	2$\times$2$\times$3 mm^3^ LSO:Ce,0.4%Ca	73	n.a.	n.a.	[41]
NINO	HPK S13360-3050CS	n.a.	n.a.	160	n.a.	[24]
NINO	FBK NUV-HD	n.a.	n.a.	135	n.a.	[24]
FlexToT v2	HPK S13360-3050CS	2$\times$2$\times$5 mm^3^ LSO:Ce,0.4%Ca	123	214	9.6	[87]
HRFlexToT	HPK S13360-3050CS	2$\times$2$\times$5 mm^3^ LSO:Ce,0.4%Ca	117	167	12.0	[24]
HRFlexToT	FBK NUV-HD	2$\times$2$\times$5 mm^3^ LSO:Ce,0.4%Ca	119	142	n.a.	[24]
FastIC	HPK S13360-3050CS	n.a.	n.a.	140	n.a.	[108]
FastIC	FBK NUV-HD LF2 M0	2$\times$2$\times$3 mm^3^ LSO:Ce,0.2%Ca	76	n.a.	n.a.	[127]
FastIC	FBK NUV-HD LF2 M0	3.13$\times$3.13$\times$20 mm^3^ LSO:Ce,0.2%Ca	127	n.a.	n.a.	[127]
FastIC	FBK NUV-MT	2$\times$2$\times$3 mm^3^ BGO	330	n.a.	n.a.	[128]
FastIC	FBK NUV-MT	2$\times$2$\times$20 mm^3^ BGO	490	n.a.	n.a.	[128]
PETA4	FBK NUV-HD	4$\times$4$\times$25 mm^3^ LYSO:Ce	460	n.a.	12.8	[23]
EXYT	SSPM	2$\times$2$\times$14 mm^3^ LYSO:Ce	363	n.a.	10.0	[102]
DIET	SensL 30020	3$\times$3$\times$16 mm^3^ LYSO:Ce	244	n.a.	11.0	[122]

### Performance

Table 4 provides an overview on important performance parameters for PET application, such as CTR, energy resolution and SPTR. Generally, using short, approx. 2$\times$2$\times$3 mm^3^, scintillators and state-of-the-art materials in terms of scintillator and SiPM, many ASICs reach a CTR in the proximity of the 100 ps performance point - some of them including the jitter of an integrated TDC. However, from the overview compiled, it is clear that comparing the performance achieved is difficult given the variety of experimental setups and materials used. Furthermore, it becomes apparent that the performance parameters reported (CTR, SPTR, energy resolution) are inconsistent among different studies. Hence, the next section aims at identifying specific steps to report the performance parameters of ASICs in PET to ease the comparability between the results for potential users.

**Fig. 9:**
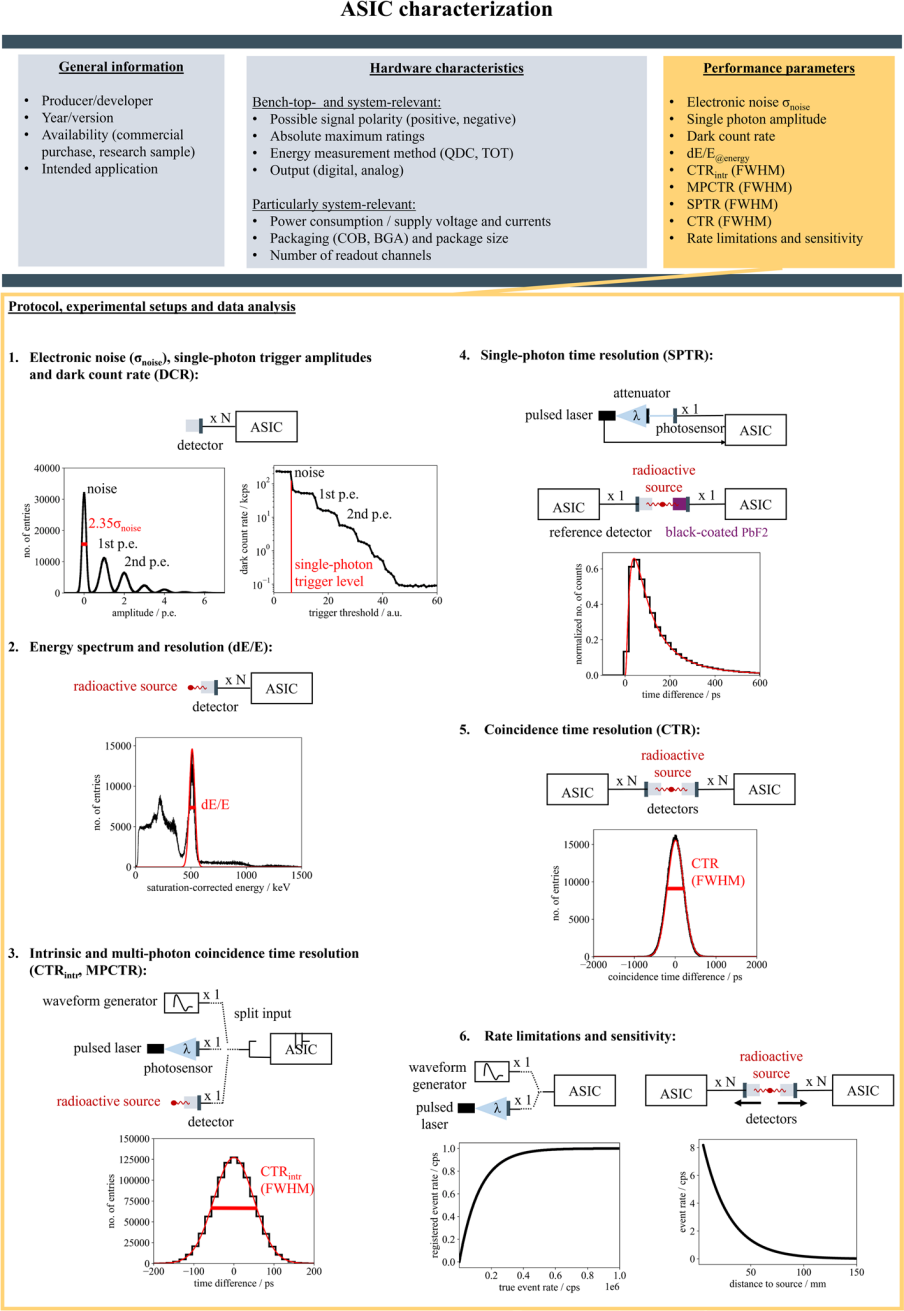
A general protocol to characterize an ASIC for PET applications

## A general protocol to characterize ASICs in PET

As the definition of the term *ASIC* and the previously introduced examples suggest, ASICs - although specifically designed for the purpose of digitizing detector signals in PET - can be very versatile. Nevertheless, this articles aims to provide some guidelines on characterizing an ASIC in the field of PET and reporting important performance parameters of the circuit to establish a consensus. Figure 9 summarizes the suggested protocol.

### Reporting general information

The authors suggest to generally report the producers or developers of an ASIC and to indicate if the ASIC is available commercially or as a research prototype. It is important to always specify the year of production and the ASIC’s version as many developers aim for highest standards and produce several iterations of their designs to implement bug fixes and new features.

Last but not least, the authors suggest to discuss the application the circuit was originally intended for. Over the past decades, the field of PET, especially the detector design and electronics, have seen major influences from the fields of HEP and LiDAR [22, 25, 86]. Hence, it is important to raise awareness among the PET research community on where and in which context to look for new developments, which potentially benefit the field of PET.

### Reporting hardware characteristics

When it comes to choosing an ASIC for their PET bench-top experiment or even a fully equipped PET scanner, many users firstly rely on the hardware characteristics of an ASIC to initially evaluate its suitability for their specific application.

The authors suggest to report the possible signal polarity (positive, negative) of the ASIC front end, the absolute maximum ratings and electrical characteristics of the input stage, both in terms of saturation, e.g., in terms of trigger rate, as well as reliability and potential damage caused by operation outside the defined specifications, e.g., feeding high current or voltage signals into the front end. Additionally, the authors suggest to provide details on the methods that can be used to measure the deposited energy (QDC, TOT) and the output of the ASIC (digital, analog). While these are characteristics considered important for all PET-related bench-top experiments as well as PET scanners, the following characteristics are considered to become particularly relevant at PET system level. The authors recommend to report the overall power consumption of the ASIC, if possible split in its analog and digital part, and the power consumption for communication with an FPGA. As discussed above, not only the different supply voltages are of importance, but also the currents drawn, especially when it comes to multi-modal imaging suffering from interference effects such as MRI (c.f. Sections "Power consumption" and "MR compatibility"). The packaging (COB, ball grid array (BGA)), the package size, the die size before packaging and the number of readout channels, etc., also normalized to the package size, i.e., the channel density, might be important characteristics to consider when it comes to system integrability.

### Reporting performance characteristics

After commenting on the influence of the PET detector configuration, this section includes a recommended protocol to characterize application-specific integrated circuits (ASICs) for PET applications.

#### Detector configuration

When talking about the performance parameters of ASICs in PET, it is crucial to understand that many of them depend to a large extend on the PET detector configuration chosen. The employed detector configuration should hence always be specified, including the producer, material composition and geometry of the scintillator, the producer, active area and SPAD size of the SiPM and any modification to the readout path before the input stage of the ASIC.

Furthermore, the authors recommend to include measurements with state-of-the-art single-pixel detectors (e.g., LYSO 2$\times$2$\times$3 mm^3^ and 3$\times$3$\times$20 mm^3^ coupled to Broadcom near ultra-violet (NUV)-metal-in-trench (MT) SiPMs [129] or Hamamatsu S14161 SiPMs [130] with an active area between 3$\times$3 mm^2^ and 4$\times$4 mm^2^). The selection of these minimize the influence of the detector geometry and enable a comparison of the performance to those achieved in other studies, allowing to estimate the impact of the ASIC on the overall performance as the detector geometry is kept in its simplest form [18].

#### Characterization protocol

The authors suggest to conduct the following steps to characterize an ASIC for PET applications and report the performance parameters listed below.

***Electronic noise, single-photon trigger amplitudes and dark count rate***

The first characterization step is related to evaluating noise, signal and trigger amplitudes. For this, the setup consists of a single-pixel detector (the usage of a scintillator is optional) connected to the ASIC input stage (see Fig. 9 step (1). If the ASIC comprises not only the analog front end but also the digitizers (see Fig. 4), it will be most practical to establish a debug output in between the analog front end and the digitizers to access the waveforms. Procedures for circuits with and without digitzers as well as with and without access to the analog signal via a debug output are introduced.

***Electronic noise***

To characterize the magnitude of the electronic noise $\sigma _\textsf{noise}$ of the ASIC front end, the user should acquire a so-called *“finger plot”* (see Fig. 9 step (1) displaying a histogram of the amplitudes of all registered events [21]. This plot can be acquired using the amplitude function of an oscilloscope or acquiring waveforms and determining the amplitudes post-acquisition using dedicated analysis software. The *“finger plot”* is expected to show peaks, of which the first peak corresponds to signals stemming from electronic noise and the subsequent peaks represent signals triggering a single, two, three or more SPAD breakthroughs in the employed SiPM. The width of the noise peak, which can be determined by fitting a Gaussian to it [54], corresponds to the magnitude of the electronic noise that has an influence on the timing jitter of the signal (see Eq. 3). The actual impact of the electronic noise on the CTR can be assessed selecting events with different deposited energies, using the Compton continuum of a 511 keV $\gamma$-interaction in the scintillator [131]. If electronic noise can be neglected, the dependence of the CTR as a function of deposited energy *E* should follow an inverse square root behavior:

7 $$\begin{aligned} \textsf{CTR}_\textsf{photostatistics} \propto \frac{1}{\sqrt{E}} \end{aligned}$$

If the electronic noise has a significant impact, the CTR becomes inversely proportional to the energy:

8 $$\begin{aligned} \textsf{CTR}_\mathsf {noise-dominated} \propto \frac{1}{E} \end{aligned}$$

For these measurements, it is important to perform a proper saturation correction, when plotting the evaluated energy (c.f. Eq. 10).

It is important to keep in mind that the employed PET detector, specifically the SiPM model with its gain and capacitance, i.e. the source impedance, will impact the outcome of the electronic noise measurement. Furthermore, all interconnections between the detector and the ASIC as well as related pick-up noise will influence the determined noise level. In addition to measurements with a PET detector, the authors therefore suggest to characterize the noise figure (NF) using a 50 $\Omega$ source impedance and to specify the input referred series and parallel noise sources [63]. Furthermore, normalization via computing the equivalent noise charge or equivalent noise current (ENC) as a measure of SNR should be considered [63, 132] and reported.

Besides electronic noise, also baseline shifts can play a crucial role in the timing performance of ASICs. Especially for high-rate applications or at elevated temperatures, an increased dark count rate of the SiPM can cause problems. Proper pole-zero corrections [133] or digital baseline corrections can be applied and it is possible to reduce this effect to almost zero in standard PET applications.

***Single-photon trigger amplitudes***

The *“finger plot”* generated to evaluate the width of the electronic noise can also be used to evaluate the amplitudes and separability of (single-)photon triggers, which are usually in the order of some dozens of mV [21]. Gaussian fits applied to the peaks representing signals triggering on a single, two, three or more SPAD breakthroughs in the employed SiPM allow to determine the centroid and the width of the peaks. The distance of the centroids and width of the peaks indicate how well different trigger amplitudes can be distinguished after the shaping of the front end and which trigger thresholds need to be applied to trigger on a specific signal amplitude. More precisely, the distance of the peaks $\Delta \mu _\textsf{peaks}$ divided by the width of the first peak $\sigma _\textsf{noise}$ represents the SNR of the readout chain:

9 $$\begin{aligned} \textsf{SNR} = \frac{\Delta \mu _\textsf{peaks}}{\sigma _\textsf{noise}} \end{aligned}$$

The latter can also be determined using a so-called *“staircase plot”* (see Fig. 9 step (1) [134]. The *“staircase plot”* shows the number of registered events at and above a certain trigger threshold depending on the threshold applied. This curve exhibits pedestals, where the first pedestal corresponds to the electronic noise and the subsequent pedestals represent signals triggering a single, two, three or more SPAD breakthroughs in the employed SiPM. Hence, the curve is similar to an integral representation of the *“finger plot”* and the transitions between pedestals, equivalent to the valleys between the peaks in the *“finger plot”*, can be used to select a trigger threshold based on the signal amplitude in terms of single or multiple incident optical photons. Differentiation of the *“staircase plot”* should furthermore enable the user to compute a *“finger plot”* without having access to a debug output, which still contains information about the distance of the trigger levels, but looses information on the acquired counts. However, a challenge is the saturation of the ASIC input. It may occur that the trigger levels applied to compile the histogram for the *“finger plot”* are not sufficiently low to resolve the noise peak. Analogously, the data rate capabilities of the ASIC may be limited and do not resolve the noise triggers as first pedestals in the *“staircase plot”*. In both cases, the noise estimate will have to be taken from the first peak or pedestal which corresponds to triggering on the first photo-electron. Showing one or both of these plots for an ASIC characterization can be essential to understanding the capabilities of the ASIC in terms of trigger levels in PET [11]. Additionally, comparing the distance of the peaks or pedestals gives information on the relative gain of the SiPM and ASIC.

It is important to keep in mind that the single-photon trigger amplitude and the ability of an ASIC to resolve single-photon triggers strongly depend on the employed SiPM with its device capacitance, microcell size as well as the configured bias voltage (c.f. Section "Detector configuration"). A way to overcome this issue and the saturation of the ASIC input stage at unknown trigger level is to employ an electrical circuit to inject a well defined electrical signal with a standardized equivalent capacitance into the ASIC front end. This would mitigate the influence of the SiPM type and properties as well as allow to understand the signal height at which the ASIC moves from noise floor to resolving pedestals.

***Dark count rate***

Both *“finger plot”* and the *“staircase plot”* (see Fig. 9 step (1) contain information about the dark count rate (DCR) of the employed SiPM, which can be defined either as the number of events in the second peak over the acquisition time or the event rate displayed at the second pedestal. While this is not a characteristic property of the ASIC itself, the knowledge of this rate can be vital to understanding the influences of noise in the PET detector.

***Energy spectrum and resolution***

Since for PET specifically interactions at an energy 511 keV are of special interest [135], the capability of the PET detector and ASIC to precisely resolve the $\gamma$-photon energy and allow for energy selection from an energy spectrum is essential. The energy values acquired using the ASIC need to be converted to keV, where one needs to account for both the non-linearity of the ASIC energy measurement method (integration, TOT) and the saturation of the SiPM [21]. Additionally, the intrinsic light yield of the chosen scintillator material, its wrapping/coating and the positioning accuracy into scintillator voxels, which can be energy-based and is in turn influenced by the energy resolution, play an important role [15, 135]. Hence, to acquire energy spectra and assess the energy resolution, the setup needs to employ a fully equipped PET detector consisting of a scintillator that is optically coupled to an SiPM with at least a single pixel, but multiple pixel to address system-relevant concerns. This correction can be performed by acquiring energy spectra with multiple radioactive sources (e.g., Cs-137, Ba-133, Co-57,...), which emit $\gamma$-photons at different distinctive energies. These peaks need to be fitted with Gaussian functions and the centroids of the peaks need to be mapped to the expected emission energy [131]. In addition to $\gamma$-emitting sources, positron-emitting sources, such as Na-22, should be considered, where the positrons annihilate with electrons and cause the back-to-back emission of two 511 keV $\gamma$-photons [40]. The curve of the expected emitted energy *E* in keV should follow a logarithmic function depending on the raw energy value *e* acquired with the ASIC, including several factors to account for corrections and saturation (*c*: linearity correction factor, *s*: saturation factor related to the number of photons detected) [21, 25, 40]:

10 $$\begin{aligned} E = s \cdot \textsf{log}(\frac{1}{1 - \frac{e}{c}}) \end{aligned}$$

Fig. 9 step 2 shows an example of such a calibrated, saturation-corrected energy spectrum in keV that was acquired using a Na-22 source and displays a distinctive peak at 511 keV, which is important to measure the energy resolution in PET. The energy resolution dE/E, commonly reported in percent, is defined as the full width at half maximum (FWHM) of the peak divided by the location, i.e., the centroid of the peak $\mu$, both determined via a Gaussian fit:

11 $$\begin{aligned} dE/E_\mathsf {@511 keV}= \frac{\textsf{FWHM}_\mathsf {@511 keV}}{\mu _\mathsf {@511 keV}} \end{aligned}$$

The same method of calculating the energy resolution may be applied at other energies [131]. It is important to state the fit range applied and whether potentially visible escape peaks were modelled or neglected.

***Intrinsic coincidence time resolution***

The intrinsic coincidence time resolution ${\hbox {CTR}}_\textsf{intr}$ characterizes the contribution of the TDC to the overall CTR. To measure this contribution, one (detector) pixel is connected to two ASIC channels and a series of identical signals is passed to both of these ASIC channels and digitized with a timestamp and energy value. Routing an identical signal to the two ASIC channels ensures that the influence of the analog front end is negligible. The identical signal can be generated using a waveform generator directly connected to the ASIC input stage. Alternatively, an SiPM can be used that either detects optical photons from a laser with an emission wavelength $\lambda$ matching the spectral sensitivity of the SiPM [71] or a single-pixel PET detector [134], i.e., a scintillator coupled to the SiPM, irradiated by a radioactive source. Coincidences are matched within a defined coincidence time window $t_\textsf{w,coinc}$ and their time differences are added to a histogram (see Fig. 9 step (3). A Gaussian fit to the histogram is used to determine the intrinsic coincidence time resolution via the FWHM of this histogram. Ideally, this parameter is stated at a trigger level calibrated to a single-SPAD signal height. If the intrinsic CTR is then evaluated with respect to a varying applied threshold, this gives a hint on the electronic noise and bandwidth [136]. Furthermore, it has to be kept in mind that the threshold level may change if two readout channels are connected to the same detector, as this effectively changes the input stage impedance seen by the SiPM [134].

***Multi-photon coincidence time resolution***

The MPCTR is measured similarly to the aforementioned intrinsic coincidence time resolution, but characterizes the time resolution contribution due to the response of the analog front end to multiple optical photons reaching the SiPM [71]. Hence, two SiPMs are each connected to an ASIC channel and irradiated by a laser with an emission wavelength $\lambda$ matching the spectral sensitivity of the SiPMs (similar to the setup sketched in Fig. 9 step 3, but with two SiPMs). The laser beam may be attenuated in such a way that the deposited charge roughly matches the charge deposition of a typical photopeak event. Still, as the photon density will strongly depend on the laser pulse width, the type of laser employed and its characteristics, i.e., specifically the pulse width, should be reported. Analogously to the steps described for the intrinsic coincidence time resolution, a coincidence time difference histogram is computed and the MPCTR is determined via the FWHM of a Gaussian fit. Here, comparing the intrinsic MPCTR, i.e., one SiPM connected to two ASIC inputs, and the true MPCTR, where two SiPMs are connected to two ASIC inputs, can give an insight into baseline fluctuations [71].

***Single-photon time resolution***

In contrast to the MPCTR, the SPTR characterizes the time resolution with which a detection chain can resolve a single incident optical photon detected by the SiPM. To reliably detect individual optical photons and differentiate them from other events, the user may employ a setup using a laser with an emission wavelength $\lambda$ matching the spectral sensitivity of the SiPMs and with the signal attenuated to single-photon level as in [47], sending a start signal to the ASIC (see Fig. 9 step (4). It is important to consider contributions of the setup to the measured time resolution, such as the timing jitter of the single-photon laser pulse as well as the synchronization signal. An alternative method, implemented and used in [49], is a coincidence setup employing a black-painted lead fluoride (${\hbox {PbF}}_2$) crystal in coincidence with a state-of-the-art PET detector (see Fig. 9 step (4). Similar to the steps described before, a time difference histogram is computed for both cases, selecting on single-photon triggers in the energy spectrum of the detector under test and, in the case of the second setup version, on 511 keV for the reference detector. The histogram of recorded time delays between “start-detector” and “stop-detector” composes the shape of the IRF of the setup, i.e., its intrinsic time resolution. The time difference histogram is fitted with either a single Gaussian function (c.f. Eq. 12, as introduced in [137, 138])

12 $$\begin{aligned} \textsf{IRF}(t) = \frac{1}{\sqrt{2}\cdot \sigma _{\textsf{IRF}}} \cdot e^{-\frac{(t-\mu )^2}{2(\sigma _{\textsf{IRF}}^2)}}, \end{aligned}$$

where $\mu$ is the mean time delay within the electronics, or a Gaussian convolved with an exponential tail, which represents photon absorption deeper in the SPAD junction (c.f. Eq. 13, as introduced in [138])

13 $$\begin{aligned} \textsf{IRF}(t) = \frac{1}{\sqrt{2\pi \sigma _{\textsf{IRF}}}} \cdot e^{-\frac{(t-\mu )^2}{2(\sigma _{\textsf{IRF}}^2)}} * \lambda e^{-\lambda t} \end{aligned}$$

The convolution can be replaced by [138]

14 $$\textsf{IRF}(t) = \frac{\lambda }{2} \cdot e^{\frac{\lambda }{2} (2\mu + \lambda \sigma ^2_{\textsf{IRF}} - 2t)} \cdot \left[ 1 - \textsf{erf} \left( \frac{\mu + \lambda \sigma ^2_{\textsf{IRF}}-t}{\sqrt{2}\sigma _{\textsf{IRF}}} \right) \right] ,$$

where $\lambda$ represents the exponential contribution of the delay tail. The error function is defined as

15 $$\begin{aligned} \textsf{erf} (t) = \frac{2}{\sqrt{\pi }} \int _0^t e^{-x^2} dx \end{aligned}$$

The FWHM of the Gaussian part of the distribution corresponds to the SPTR. Of course, if this evaluation is to be performed, the circuit should be able to resolve the aforementioned single-photon trigger amplitudes for event selection. It should always be explicitly stated if the reported value was corrected for electronic noise [47]. The SPTR parameter is critical in low-photon count detection, e.g., Cherenkov emission, but becomes less influential in multi-photon detection.

***Coincidence time resolution at 511 keV***

The CTR of a readout chain corresponds to the TOF resolution in a TOF-PET system (neglecting system effects, e.g. event positioning and scatter rejection) and, thus, is one of the most important parameters for PET. It is determined using a setup of two state-of-the-art PET detectors in coincidence and a positron-emitting radioactive source to generate two coincident 511 keV $\gamma$-photons, whereby multiple compositions (single pixel with shorter and longer scintillators and entire detector blocks) should be evaluated to estimate the influence of physical effects such as PTS, light-sharing and crosstalk on the CTR. Similar to the setups before, a coincidence time difference histogram is computed and fitted with a Gaussian function. The FWHM of this distribution corresponds to the CTR (see Fig. 9 step (5). In HEP applications, it is common to report the time resolution as RMS ($\sigma$), which should be converted to FWHM using

16 $$\begin{aligned} \textsf{FWHM} = 2\sqrt{2\log (2)}\sigma \approx 2.35\sigma \end{aligned}$$

In case a reference detector is used, the measured CTR needs to be corrected for the coincidence time resolution of the reference detector $\textsf{CTR}_\textsf{ref}$ via

17 $$\begin{aligned} \textsf{CTR} = \sqrt{2\cdot \textsf{CTR}_\textsf{meas}^2 - \textsf{CTR}_\textsf{ref}^2} \end{aligned}$$

It is important to state the energy filter applied to the events. It is recommended to use a filter of $\pm 2\sigma$ around the photopeak. For clinical applications, the evaluation benefits from additionally stating the performance in different energy windows in keV [57].

***Rate limitations and sensitivity***

To investigate the maximum event rate that an ASIC allows to digitize, the events need to be generated with a defined rate. This can be achieved by directly connecting a waveform generator to the ASIC input stage or by irradiating an SiPM with a pulsed laser at a defined pulse frequency [71]. A curve plotting the registered events against the true event rate, as shown in Fig. 9 step 6, is expected to show a saturation for the events registered. Here, it is important to consider the limitations of the ASIC readout, e.g., input and output buffer of the employed FPGA. Sensitivity tests can be conducted on bench-top level varying the distance of a radioactive source to the detector and computing the geometrically expected rate in relation to the measured rate [120]. For this an experiment with two single-pixel detectors, but also two detector blocks (aiming to equip all channels of one ASIC) should be conducted plotting the acquired coincidences versus the distance to the source, as shown in Fig. 9 step 6.

Generally, it is important to note that the maximum event rate does not solely depend on the ASIC itself, but can also be limited by other components of the setup used, such as an integrated FPGA or transfer rate limits when writing to the data acquisition and processing server. Furthermore, it has to be kept in mind that high data rates can cause baseline fluctuations at the ASIC input, especially in the case of an AC-coupled signal path. In a high-rate regime, where the baseline is unable to recover before the next trigger arrives, this may have significant impact on the multi-photon and coincidence time resolution [71], as it adds additional jitter on the trigger point of leading-edge discriminators. Signal filters can be employed to stabilize the baseline in these cases [133].

## ASICs and computational intelligence

The digitized output of an ASIC, i.e., timestamp and energy values, is typically processed with analytical methods as described in the characterization protocol in Section "Characterization protocol". However, in recent years, many attempts to simplify these calibration procedures and performance assessments have been published using data-driven methods like machine learning (ML).

The use of ML techniques has transformed various fields. Machine learning systems can learn from presented data using algorithms and statistical models that can handle multidimensional data. Without loss of generality, we will refer to a trainable learning system as artificial neural network (ANN) in the following.In recent years, the impact of machine learning on science has been profound. It has accelerated research and discovery by automating data analysis, enhancing predictive modeling, and enabling the processing of vast amounts of data with increased speed and accuracy. One notable area where machine learning has made significant strides is in medical imaging. In PET, computational learning methods are applied to a wide range of applications, from image reconstruction and analysis to processing raw detector signals or even signal compression [139–141]. While current research in PET mainly uses conventional hardware platforms (central processing units (CPUs) and graphical processing units (GPUs)) for training and inferring of ANNs, there is also research focusing on hardware-embedded (on-chip) applications of artificial learning systems aiming to provide online processing abilities [142].

Typical ASIC outputs that are used as input for ANN applications in PET are mostly related to information about the detection of $\gamma$-photons using SiPMs. Depending on the targeted use case, this output information could be the integrated signal charge of one or many SiPMs, pulse amplitudes or waveform information of the detection signal, and also the measured timestamps.

### Offline applications of ANNs

There exists a wide variety of research studying the offline application of ANNs in PET, which is characterized by the fact that the inferring of trained models is done on CPU or GPU. With regard to the application of ANNs to ASIC output, typical PET-based use cases are the estimation of the $\gamma$-interaction position (depth of interaction (DOI)) inside the scintillator or TOF-related predictions aiming to improve the achievable CTR and hence localize the position of a radioactive decay along the line of response (LOR). Encoding the DOI has a direct impact on the spatial resolution of a PET system, while a high TOF resolution, i.e., a high CTR, improves the SNR of the PET image. Assuming TOF resolution in the sub-100 ps range, both have an impact on the voxelization for PET reconstruction. Depending on the available use case, different kinds of input data have to be acquired, and different network topologies are used.Typically, for estimating the $\gamma$-interaction in a scintillator volume, the digitized energy signals from the underlying SiPMs are accessible from the ASIC and used as input for the neural network. Recent research demonstrated the feasibility of this approach utilizing multi-layer perceptron (MLP) [143] or convolutional neural network (CNN) [144] architectures.

Concerning machine learning research targeting TOF improvement many studies make use of the waveform signals [145–149] as input for CNN-based architectures. However, with the currently available ASICs, waveform sampling is not feasible due to limitations in the sampling rate. In a simulation study [150], a CNN approach was tested using multiple timestamps derived from a waveform signal instead of the full waveform. Recently, TOF improvement was shown using timestamps of multiple SiPMs that had been digitized by the PETSys TOFPET2 ASIC in combination with gradient-boosted decision trees (GBDT) [151]. The authors applied a residual-physics-based approach [152, 153] in combination with gradient-boosted decision trees (GBDT) to clinical detector stacks.

### Hardware-embedded applications of ANNs

Especially for cases where high data rates are expected, and real-time processing is desired, it is essential to consider implementing ANNs in the hardware (Edge-AI [154], gradient tree boosting in FPGA [155]). However, implementing ANNs in front-end electronics involves significant challenges, including limited power budgets, dense space requirements, and extreme operational conditions such as high radiation and cryogenic temperatures [156]. Many developments in this field of research have been driven by HEP applications comprising a large number of detector elements and need for hardware-embedded solutions due to enormous data rates, e.g. by using an autoencoder network for data compression at the large hadron collider at CERN [157].However, recent works from the field of PET demonstrated the interest of the PET community for on-chip ANN research. In [142], a fully reconfigurable on-chip feed-forward ANN was presented aiming to reconstruct the radiation source position between two detectors. Four processors synthesized through highlevel synthesis (HLS) accelerate the ANN operations, managed by a control logic unit coordinating the sequential execution steps. The reconfigurability of the ANN is achieved by the possibility of changing the network topology within the limits of the design of a feed-forward network and hardware. At the maximum, the implementation supports up to 128 neurons, along with 1024 weights and biases. In their work, the authors showed the functionality of the approach by using TDC codes as input for the network, which had previously been trained offline using the PyTorch framework. The concluded information about the topology, weights, and biases are then used for the configuration of the on-chip ANN via an AXI bus. The authors claim an efficiency of 190 GOP/s/W.

In another work [158], an on-chip ANN implementation is studied, which aims to reconstruct the $\gamma$-interaction position inside the scintillator of Anger cameras used in PET and single-photon emission computed tomography (SPECT). The computation is performed in the charge domain using crossbar arrays of programmable capacitors. In the study, the authors use the 64 output signals of the 8 $\times$ 8 SiPMs as input. Furthermore, the network comprises two hidden layers with 20 neurons each, and two output signals ranging from 0 V to 3.3 V encoding the *x*, and *y* value of the interaction point. Before the on-chip application, the ANN is trained offline in MATLAB using weight quantization (QAT). The estimated efficiency is about 93.5 GOP/s/W.

## Future perspectives

With the trend to decrease the form factor of electronic devices seen over the past decades and the manufacturers’ capability to implement smaller and smaller CMOS processes below the 100 nm scale, we expect the form factors of ASICs to decrease as well. Advances in packaging and vertical integration techniques will become of paramount importance to achieve high performance and high channel density at the same time. This goes in line with requirements for even lower power consumption and lower heat dissipation as well as adaptability of the power and current consumption in software, all especially important for hybrid system integration. Integrated cooling techniques, pipes or micro-channels will be essential to achieve this goal. The increased demand for extracting more information from a signal, e.g., multi-thresholding or even waveform sampling, however, may diverge from this development and pose a challenge to the implementation of small-area chips.

While the aforementioned parameters are subject to the ASIC itself, the field might turn the spotlight on the interface between the ASIC and residual readout infrastructure to achieve further channel compression, smaller form factors and higher flexibility at the same time. Conceivable are common definitions of data transfer protocols at the interface between ASIC and FPGA, programmable matrix definitions of interconnections for multiplexing as well as daisy-chain concepts for data and power links to connect several ASICs.

Generally, ASICs are expected to be adaptable to the latest trends in PET applications, such as scanners with an enlarged aFOV, leading to an increased number of channels and an overall higher amount of data. At the same time, other fields of medical imaging, such as CT or radiation therapy would benefit from radiation-hard ASICs capable of processing high data rates. Here, translation of developments in HEP may play a crucial role.

Last but not least, the detector concepts in PET are currently being rethought, which might lead to a transition from multi-photon to single-photon detection regimes, causing a shift in performance parameters to be optimized. With the aim to digitize Cherenkov photons from mixed or pure Cherenkov emitters, the single-photon detection capabilities of ASICs need to be focused on and the SPTR needs to be taken into account as the most important performance parameter. Furthermore, employing scintillators with a lower expected production cost, such as BGO, the ASIC design and cost are required to be kept low as well to avoid the electronics becoming the most cost-expensive component of the readout chain.

## Conclusion

This article provides an overview on existing and emerging readout solutions in PET over the past 20 years, which have shown a trend towards lower power consumption, smaller form factors and higher numbers of readout channels in highly integrated ASICs, including or excluding digitizers. The field of ML is conquering the field of signal digitization and processing, facilitating the calibration and processing for analog or discrete waveforms as well as already digitized data. This study shows that due to the versatility of the readout solutions available, which are highly adapted to the employed detector concepts, it is very difficult to compare their performance and evaluate their potential for further developments in comparison to other ASICs. To tackle this challenge, a general protocol to characterize ASICs for PET applications has been compiled. Furthermore, the authors have formulated a wish list on desired characteristics and values for the ASIC performance parameters. The authors conclude that the community of ASIC developers should continue to strive for the specified goals and further advancements in their fields. At the same time, the users of the ASICs selected for PET applications are required to thoroughly investigate their performance according to the protocol specified and provide well-founded feedback to the ASIC developers for them to detect and overcome limitations.

## Data Availability

Not applicable.

## Abbreviations

ADC: Analog-to-digital converter
aFOV: Axial field of view
ASIC: Application-specific integrated circuit
ANN: Artificial neural network
HLS: High-level synthesis
BGA: Ball grid array
BGO: Bismuth germanate, ${\hbox {Bi}}_4{\hbox {Ge}}_3{\hbox {O}}_{12}$
CERN: European Council for Nuclear Research (Conseil Européen pour la Recherche Nucléaire)
CIEMAT: Centre for Energy, Environmental and Technological Research (Centro de Investigaciones Energéticas, Medioambientales y Tecnológicas)
CMOS: Complementary metal-oxide-semiconductor
CMS: Compact myon solenoid
CNN: Convolutional neural network
CPU: Central processing unit
COB: Chip on board
CSP: Chip scale package
CT: Computed tomography
CTR: Coincidence time resolution
DCR: Dark count rate
DLL: Delay-locked loop
DOI: Depth of interaction
EEG: Electroencephalogram
ENC: Equivalent noise charge or equivalent noise current
FBK: Fondazione Bruno Kessler
FOV: Field of view
FPGA: Field-programmable gate array
FWHM: Full width at half maximum
GBDT: Gradient-boosted decision trees
GPU: Graphical processing unit
HEP: High energy physics
HF: High-frequency
ICCUB: Institut de Ciènces del Cosmos at University of Barcelona
ILY: Intrinsic light yield
LiDAR: Light detection and ranging
LOR: Line of response
LTE: Light transfer efficiency
LVDS: Low voltage differential signaling
LSO: Lutetium oxyorthosilicate, ${\hbox {Lu}}_2{\hbox {SiO}}_5$:Ce
LYSO: Lutetium yttrium oxyorthosilicate, ${\hbox {(Lu-Y)}}_2{\hbox {SiO}}_5$:Ce
MCP: Micro-channel plate
ML: Machine learning
MLP: Multi-layer perceptron
MR: Magnetic-resonance
MT: Metal-in-trench
MRI: Magnetic resonance imaging
MPCTR: Multi-photon coincidence time resolution
NF: Noise figure
NUV: Near ultra-violet
PbF2: Lead fluoride
PCB: Printed circuit board
PDE: Photon detection efficiency
PDH: Peak detector and hold
PET: Positron emission tomography
PTS: Photon time transfer spread
QDC: Charge-to-digital converter
RF: Radio-frequency
RMS: Root mean square
SiP: System in package
SiPM: Silicon photomultiplier
SNR: Signal-to-noise ratio
SPAD: Single-photon avalanche diode
SPECT: Single-photon emission computed tomography
SPTR: Single-photon time resolution
TDC: Time-to-digital converter
TOF: Time-of-flight
TOT: Time-over-threshold
